# Dynamics of extinction debt across five taxonomic groups

**DOI:** 10.1038/ncomms12283

**Published:** 2016-07-25

**Authors:** John M. Halley, Nikolaos Monokrousos, Antonios D. Mazaris, William D. Newmark, Despoina Vokou

**Affiliations:** 1 Department of Biological Applications and Technology, Faculty of Health Sciences, University of Ioannina, 45110 Ioannina, Greece; 2 Department of Ecology, School of Biology, Aristotle University of Thessaloniki, 54124 Thessaloniki, Greece; 3 Natural History Museum of Utah, 301 Wakara Way, University of Utah, Salt Lake City, Utah 84108, USA

## Abstract

Species extinction following habitat loss is well documented. However, these extinctions do not happen immediately. The biodiversity surplus (extinction debt) declines with some delay through the process of relaxation. Estimating the time constants of relaxation, mainly the expected time to first extinction and the commonly used time for half the extinction debt to be paid off (half-life), is crucial for conservation purposes. Currently, there is no agreement on the rate of relaxation and the factors that it depends on. Here we find that half-life increases with area for all groups examined in a large meta-analysis of extinction data. A common pattern emerges if we use average number of individuals per species before habitat loss as an area index: for mammals, birds, reptiles and plants, the relationship has an exponent close to a half. We also find that the time to first determined extinction is short and increases slowly with area.

## Abstract (web summary)

Extinction after habit loss does not occur immediately. Here, the authors develop a model and estimate how fast extinction debt is paid off after habit loss, and show a temporal profile of species diversity decays in a power-law fashion with a half-life increasing slowly with habit size and area.

## Graphical abstract

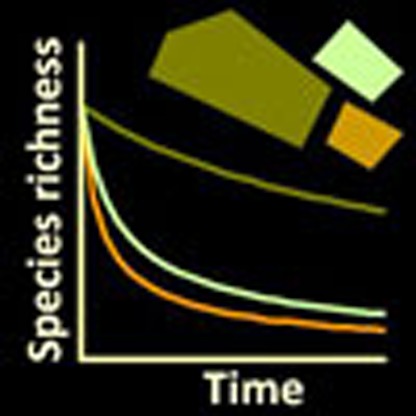

Loss of natural habitats is one of the major environmental problems of our time and with it comes the danger of irreversible loss of biodiversity. Due to human activities, many previously continuous habitats have now been reduced to fragments. These habitat remnants are left with levels of biodiversity unsustainable in the long run. Extinctions follow but not immediately^1^,^2^,^3^. The biodiversity surplus or extinction debt^4^,^5^,^6^ is lost with some delay^7^,^8^ through the process of relaxation^1^. The rate, at which it happens, and the factors that it primarily depends on, are still controversial issues. Nevertheless, it is crucial for conservation^2^,^3^,^6^ to estimate the time constants of relaxation.

Since extinction is difficult to observe, theoretical approaches have an important role to play. To describe relaxation, a negative exponential decay has frequently been assumed^1^,^2^,^6^. In such a model, species are effectively independent of each other and extinction is associated with the environmental factors rather than community interactions. An alternative form is the negative hyperbolic decay^9^,^10^,^11^,^12^, which can be associated with various ecological mechanisms, such as competition^9^ or neutrality^12^. In this model, species density plays a role, so that extinction rate slows markedly once most of the extinction debt has been paid. Such a relaxation curve may also be derived from the neutral model^12^, which assumes all individuals from all species to be equivalent, and demographic stochasticity to be the only force at work in a fixed and finite environment^13^. Because of these non-biologically grounded assumptions, the neutral model has been criticized^14^,^15^. Nevertheless, it yields an explicit relationship of half-life (*t*_50_) with area, population density, generation time and initial species richness. It has also provided reasonable predictions when applied to birds^12^. More comprehensive comparisons of theoretical estimations with real data can determine which of these models gives the best fit and which ecological factors affect the relaxation process.

A considerable number of studies have examined relaxation time, which is the time required for the extinction debt to be paid off. The timescales for this to happen have been reported to range from a few years^3^,^16^ to thousands of years^1^,^9^. There is still no consensus on the determinants of relaxation time. Several factors are at work: population size, metapopulation processes as well as the ongoing patterns of habitat loss^17^. Most argue that extinction debt is paid faster in smaller habitat remnants^1^,^2^,^9^,^12^,^18^, but others argue that the relaxation time is insensitive to area, except at extremely small sizes^6^,^19^. There have even been claims that diversity declines faster on larger islands^16^ or that extinction debt may be entirely absent for certain community types^20^.

From a conservation perspective, the delay of extinction following habitat loss offers a critical opportunity for action. If habitat restoration is accomplished before species start to disappear, irreversible damage to the community can be forestalled^2^,^6^,^21^. Thus, it is of paramount importance to know how large the delay is, the shape of the decay curve (Fig. 1) and the factors they depend on.

Here we carry out an extensive meta-analysis of published data on extinction debt to interpret existing observations and predict patterns of response. We explore the dependence of the relaxation rate on fragment area, species richness and population abundance and we refine the existing models of biodiversity decay. We do this separately for different taxonomic groups and we find that the half-life of extinction debt increases with area for all groups examined. If we use average number of individuals per species before habitat loss as an area index, the relationship has an exponent close to a half, irrespective of large taxonomic differences. We also find that the time to first determined extinction is short and increases only slowly with area. On the basis of the best-fitting model, the species decay in time is not exponential but a power law, suggesting that species loss occurs over a wide range of timescales.

## Results

### Theoretical predictions for relaxation rates

For the rate of biodiversity decay, we develop a model that combines an existing model for extinction^9^,^10^,^11^ with the neutral model^12^. An important measure of the vulnerability to extinction is the average number of individuals per species before habitat loss:


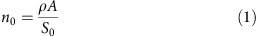


Here *A* is the area of the habitat remnant, *ρ* the density of individuals over all species of the community studied and *S*_0_ the initial species richness. In this paper, we use *n*_0_ as a proxy for area. With this rescaling of area, the solution of the model, equation (6) (see Methods; Supplementary Note 1), can be used to find the time constants (in generations) that describe the rate of decline in species richness. For the half-life of extinction debt, we have:


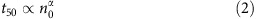


For the time to the extinction of the first species (*S*_0_→*S*_0_-1), we have:


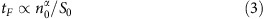


The values that *t*_F_ takes are obviously smaller than *t*_50_. A third time constant of interest is *t*_L_, which describes the time to the last determined extinction associated with the relaxation process (Supplementary Note 2). In cases where the initial species number *S*_0_ of a fragment is not known, or when making forecasts for the future, we can use the continental species–area relationship (SAR) for *S*_0_ (Supplementary Note 3).

### Meta-analysis of relaxation observations

To find *α* and the constant of proportionality associated with equation (2), we carried out a meta-analysis of a large number of published data sets of extinction data and compared the decline of species richness with the solution of the model (see Methods and Supplementary Notes 4 and 5). The analysis covered the taxonomic groups for which there were enough data suitable to parameterize our model. These were mammals, birds, reptiles, invertebrates and plants (Table 1; Supplementary Table 1).

**Figure 2:**
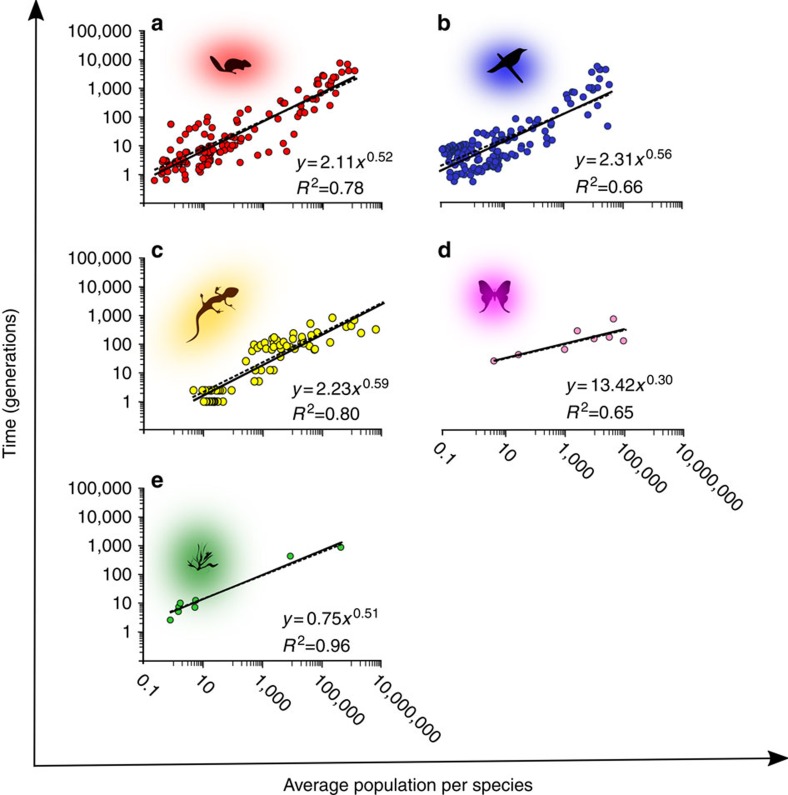
Dependence of half-life on habitat remnant area. Each point represents the estimated half-life for a single fragment using the formula for the neutral-community half-life, equation (9). Each solid line is a direct regression fit to these points while the dotted lines are the best-fitting models using the full nonlinear regression approach (Supplementry Equation 14).These figures are equivalent to Fig. 3b in Halley and Iwasa^12^; only we use as area index the average number of individuals per species before habitat loss, *n*_0_=*ρA*/*S*_0_. The different panels are for (**a**) mammals, (**b**) birds, (**c**) reptiles, (**d**) invertebrates and (**e**) plants. Note that here exponents and *R*^2^ refer to the direct regressions, not the nonlinear ones of Table 1.

We find extinction debt to be a universal phenomenon; in almost every case, we observe a delay in the extinctions following loss of habitat. For all groups (Fig. 2), we find a significant increase of half-life, *t*_50_, with area index *n*_0_ (and also with area alone), so the estimated exponent *α* is always positive. It is also fairly close to *α*=1/2, with the exception of invertebrates, for which a weaker relationship (*α*=0.31) is found. The strength of this pattern is underlined by our sensitivity analysis (Supplementary Fig. 3). The intercept in Fig. 2 (value at *n*_0_=1) can be understood as the average number of generations to extinction, when *n*_0_=1, that is, when the average area per species can only support one individual. Clearly, when initial populations are this small, we expect extinction within a small number of generations. Again, this is what we see for all taxonomic groups except invertebrates, for which the intercept is substantially higher. Invertebrates would seem to constitute an outlier to the pattern. However, we assign less confidence to the parameters that we used for our model from the invertebrate studies for a number of reasons. For example, some studies look only at the most common species while others employ morphospecies as a surrogate for species (see Methods). If we combine the results for all taxa except invertebrates the result is:


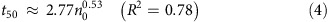


**Figure 3:**
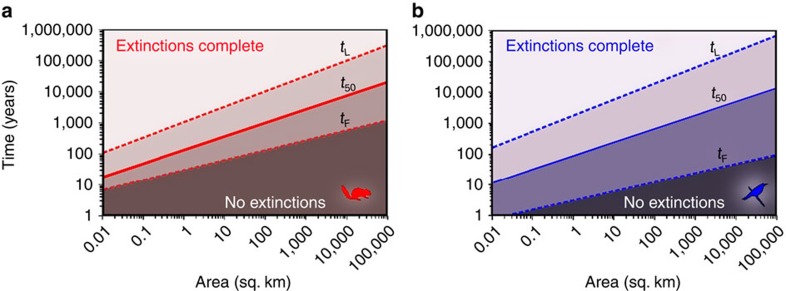
Time constants associated with the relaxation process in isolated fragments. These are given for two taxonomic groups: (**a**) small mammals and (**b**) tropical forest birds. The unbroken line is that of *t*_50_ as predicted by Supplementary Equation 9. The lower broken line is the expected time to first extinction, *t*_F_, according to Supplementary Equation 10. The upper broken line is the expected time to last extinction *t*_L_ (Supplementary Equation 11). The calculations were based on appropriate parameters *S*_0_, *ρ* and *τ* for these two taxonomic groups (see Supplementary Note 3).

To make predictions using equation (4), we need to know the species number and the average number of individuals per species before habitat loss. In the absence of direct knowledge of *S*_0_, the continental Arrhenius SAR can be used to describe the community before habitat loss. In Fig. 3, we use this approach to examine how the time constants, *t*_F_, *t*_50_ and *t*_L_, change with area for two of the most commonly studied groups: small mammals and tropical birds. All three constants increase with area but with different exponents. For example, a habitat remnant of 10 km^2^ for birds has a half-life of 351 years, but the first extinction happens much sooner, in <10 years. In Supplementary Note 3, we show that for a SAR with exponent *z*, the half-life and time until first determined extinction is related to area as *t*_50_∼*A*^*α-zα*^ and *t*_F_ ∼*A*^*α-z*(1+*α*)^, respectively, so that if *α*=0.5 and *z*=0.15, then *t*_50_∼*A*^0.425^ and *t*_F_∼*A*^0.275^. Thus, the time to first determined extinction, an important threshold for conservation action, increases only slowly with area. Even for a 1,000 km^2^ area, the first extinction is expected to happen in just 32 years. This means that the delay in extinction is not large enough to constitute a policy excuse to ‘kick the can down the road' to the next (human) generation.

## Discussion

Because of its central role in the dynamics of biodiversity loss, the time for half of the extinction debt to be paid off, *t*_50_, is the natural unit through which to parameterize the model. However, in conservation biology, the time until first determined extinction, *t*_*F*_, is also of importance. This is because it represents the time before relaxation commences, whereas at *t*_50_ the relaxation process is already half-completed. Given this, the time until first determined extinction marks an important threshold for conservation action^6^ to forestall extinctions by re-establishing connectivity among habitat remnants and restoring degraded land.

The expected time to first determined extinction is also very useful in the design of surveys related to extinction debt. For the example on birds used in Fig. 3, the first extinction happens in ∼10 years. Therefore, a search for biodiversity loss is not likely to give a signal before this time. On the other hand, if the habitat remnant has been isolated for thousands of years, the current species richness will say nothing about the isolation event because all extinctions caused by that event will have been completed. For a survey to yield useful information about the relaxation process, we should have *t*_F_<Δ*t*<*t*_L_.

For the trajectory of species loss in time, negative exponential^1^,^2^,^6^ and power-law forms^9^,^10^,^11^ have commonly been used. A direct comparison of these two forms would require observations of relaxation trajectories, which are not available, apart from a very few studies (for example, Ferraz *et al.*^3^). Our assumed general form of Supplementary Equation 4 allows for both power-law and exponential solutions for the dynamics of relaxation. If *α*>0, the solution approaches a power-law form when Δ*t* is large. An exponential pattern of relaxation can result in cases where *α*=0. However, for all taxonomic groups, the values of *α* that we estimate from the data lie well above zero and so our results strongly suggest a power law rather than an exponential pattern of decay. When relaxation follows a power law, it is initially rapid but becomes much slower thereafter; extinctions are distributed more evenly across timescales rather than dominated by a single timescale.

The results of our approach underline the widely appreciated fact that habitat loss is a key factor in species extinction^22^,^23^. They also demonstrate that, regardless of differing ecological histories, different taxonomic groups can respond similarly to a reduction in population size associated with reduction of their habitat area and that we can assign characteristic rates of biodiversity decline using only area, initial species richness, average per species population density and average generation time.

## Methods

### Population-based model

For the decay of species richness following the loss of area, we use the model^9^,^10^,^11^,^12^:


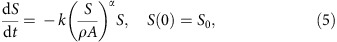


Here *S* is the species richness in the habitat remnant, *t* is the time since area loss, *A* the area of the habitat remnant, *ρ* the density of individuals, while *k* and α are constants. Equation (1) can be solved by direct integration to yield the following equation (Supplementary Note 1):


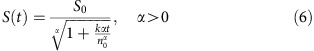


where *S*_0_ denotes initial species richness. We can show that the time required for *S*(*t*) to fall to half of its initial value is:


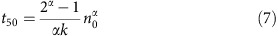


Equation (6) can also be used to find the time constants *t*_F_ and *t*_L_ (Supplementary Equations 7 and 8). For this model, habitat loss is assumed to be sudden, complete and permanent, which means that there is no life supported in the matrix between islands^24^, no re-growth of forest^25^ and no restoration of habitat. It is also assumed that the area of the habitat remnant is much smaller than the initial area and that subsequent changes in its size or isolation are negligible.

### Fitting the model to observations

Suppose that in a time Δ*t* after habitat loss, species richness has fallen from *S*_0_ to *S*_2_. Equation (6) together with equation (7) can be re-arranged to provide the estimated half-life, *T*_50_, on the basis of the observed data:


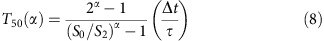


Here *τ* is the generation time. Comparing the estimated half-life and that predicted by the model, we try to find the values of *α* and *k* that minimize the difference between them. Since *T*_50_ is itself a function of *α*, this calculation is somewhat inconvenient. For example, it requires nonlinear solution techniques and is not easily visualized (Supplementary Equation 14). However, in Fig. 2, we see that the exact model fitted by nonlinear regression to *T*_50_(*α*) is in fact very close to the simple regression line fitted to the estimated neutral half-life *T*_50_(1).


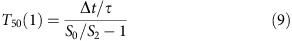


The fitting is done in the logarithmic domain because there is a large range of scales involved (Supplementary Fig. 2). A sensitivity analysis (Supplementary Note 6; Supplementary Fig. 3) shows the relative effect of uncertainty in each of the input parameters.

### Use of published data

To find *α* and the constant of proportionality associated with equation (2), we compared the decline of species richness with the solution of the model (6), as found in different studies of extinction in habitat remnants. Usable studies were those from which we could extract (or infer) Δ*t*, *S*_0_, *S*_2_, *ρ* and *A* as well as the generation time *τ*. Overall, we found more than a hundred empirical studies from 1971 (ref. 26) to the present (Supplementary Table 1) examining extinction debt, but there were only 43 from which we could extract the necessary information or supply the missing bits ourselves. These yielded a total of 385 observations that we used to parameterize the model. We limited our analysis to taxonomic groups for which we could find at least three independent studies. These were mammals, birds, reptiles, invertebrates and plants (Table 1; Supplementary Table 1).

To use a source for our analysis, it should satisfy to a large degree the requirements presented above for rapid, complete and permanent isolation of habitat remnants, and provide at least two reasonably accurate observations of biodiversity, *S*_0_ (close to the time of habitat loss) and *S*_2_, current area (*A*), and the time elapsed (Δ*t*) since habitat contraction. A source could be still used even when these parameters were not given explicitly, if they could be calculated. *S*_0_ is usually the most problematic of these parameters. If not given by the authors of the study, we assumed that the initial species number is equal to the ensemble of current species from all islands or fragments. We did not formally distinguish between estimates of *S*_2_ based on surveys or sampling. Sometimes, Δ*t* was not so clearly defined. In such cases, we took an average value between the maximum and minimum plausible times of habitat loss. The fragment area *A* was usually explicitly given.

In addition to parameters specific to individual fragments, we need to know the two parameters specific to the community: generation time *τ* and population density *ρ*; *τ* is in years and *ρ* is in individuals per hectare. Values for both parameters were taken either from the original source or other relevant literature.

Most studies of birds that we used were carried out in the tropics, for which a number of studies identified *ρ* to be close to the value used by Halley and Iwasa^12^, which we also follow here (*ρ*=16.58 per hectare). For *τ,* we use the value 5 years^14^. For mammals, because body size varies widely, we grouped species into two broad categories, ≤0.5 and >0.5 kg, and derived category-specific values of *τ*; the latter were based on allometric relations between age of maturity^27^ and generation time^28^. We derived *ρ* on the basis of species- and category-specific estimates of population density^11^,^29^,^30^,^31^,^32^,^33^. A total of four studies gave data on reptiles, most of which are exclusively or mostly on lizards; the value *τ*=2.46 that we use for all originates from Hairston^34^. Regarding *ρ*, we use the values deduced by Buckley and Jetz^35^: *ρ*=1,920 per hectare for islands, but for Singapore, which is technically an island but not well isolated, we use the mainland value, *ρ*=128. In a study undertaken at the Thousand Island Lake, in China^36^, authors provide population densities for each species that they observe; the value *ρ*=84.31 that we use is the overall density of the lizard community found from the sum of these population densities.

In our study, invertebrates are the most problematic group, not only because of their large species numbers and wide ranges of *τ* and *ρ*, but also because of the small number of usable studies and the frequently incomplete sampling. Two studies of butterflies that we made use of were in the tropics. For *τ*, we use the average estimated by Grøtan *et al.*^37^. Density of butterflies is difficult to estimate as they are very patchily distributed. Nevertheless, a density of 10^4^ per hectare is a plausible value (M. Weimers, personal communication). For Amazonian beetles^38^, it is estimated to be *ρ*=4.03 × 10^4^. There is little work existing on the generation time of tropical forest beetles. A study for a single species^39^, the Indonesian lady beetle (*Epilachna vigintioctopunctata*), estimates a generation time of 59 days (*τ≈*0.16 years). Values of several weeks are common for bark beetles in New Guinea, whereas 6 months is common for smaller- and mid-sized cerambycids (V. Novotny, personal communication). On this basis, we assigned *τ* a value of 0.16 for this class of beetles. We can estimate the density of microarthropods in a straightforward way to be *ρ*=4.64 × 10^8^ per hectare, from figures in Gonzalez and Chaneton^40^. For generation time, we arrive at a value of *τ=*0.075 years^41^,^42^. Species identification is an issue for invertebrate studies. For example, Didham *et al.*^38^ consider only the 29 most common species and Gonzalez and Chaneton^40^ only consider operational morphospecies.

Three studies give results for plants usable in our model. Leigh *et al.*^43^ provide all the parameters explicitly: a value of *τ*=30 for generation time, whereas for density a separate *ρ* is given for each of the six islands studied. For the tropical forest of Singapore^8^, we used the average density of forest plants^44^,^45^ for the Amazonian Ecuador and Brunei: we took the average of three values (*ρ*=6,568). Because most herbs are perennial in tropical forests^44^, we used a value of *τ*=3 years. The study by Drayton and Primack^46^ was conducted in a mixed forest in the Boston area of the United States. As it provides neither *ρ* nor *τ*, we estimated them in the following way: Pearson *et al.*^47^ give an estimate of 18.33 individuals per 0.5 m^2^ in less dense plots and 31.66 in denser plots for forest fragments in North Carolina. We took the average of the two, which corresponds to *ρ*=5.0 × 10^5^ per hectare. This also agrees with the results of a long-running study in Ioannina University campus, Greece (a similar biome), conducted by the authors of this study, finding a plant community density of 3.65 × 10^5^ stems per hectare in mixed forest. For generation time, we use *τ*=1 because most of the plants involved are annual herbs.

For further details of the methods, see Supplementary Notes.

### Data availability

All sources with extinction data as well as all specific data used from these sources are listed in Supplementary Table 1. In Methods and Supplementary Notes, we provide information on either the additional sources that we used to find missing data needed for our calculations or the methods that we used to derive them. Any other relevant data are available from the authors on request.

## Additional information

**How to cite this article:** Halley, J. M. *et al.* Dynamics of extinction debt across five taxonomic groups. *Nat. Commun.* 7:12283 doi: 10.1038/ncomms12283 (2016).

## Supplementary Material

Supplementary Information Supplementary Figures 1-3, Supplementary Table 1, Supplementary Notes 1-6 and Supplementary References

## Figures and Tables

**Figure 1:**
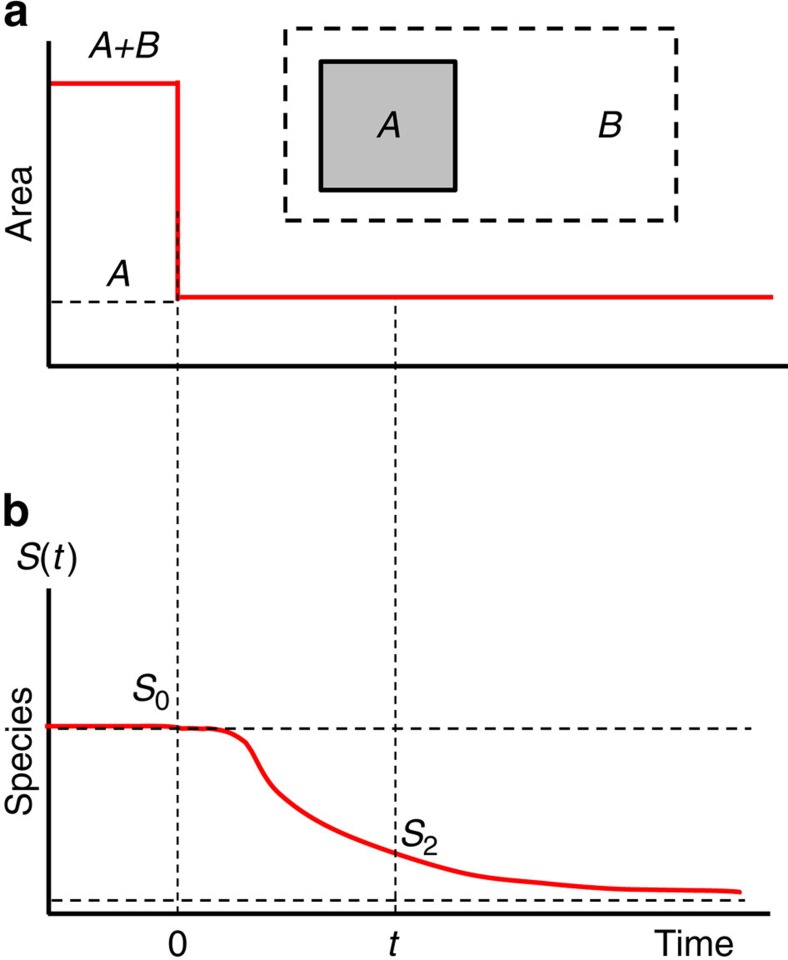
Relaxation process assumed for a habitat remnant. At time *t*=0, an area *B* is lost (**a**) leaving only the area *A* (top, inset). Following this, the species richness (**b**) relaxes to a new lower equilibrium. The area loss is assumed to be total and permanent.

**Table 1:** Estimation of parameters of the best-fitting models.

**Taxon**	**No. of points**	***α***	***k***	**Intercept**	***R***^**2**^	**No. of sources**
Mammals	129	0.49±0.06	0.21±0.07	3.99	0.77	10
Birds	161	0.52±0.05	0.29±0.10	2.89	0.66	19
Reptiles	71	0.59±0.22	0.33±0.21	2.61	0.79	4
Invertebrates	7	0.31±0.11	0.07±0.03	11.72	0.64	4
Plants	8	0.48±0.18	0.92±0.55	0.9	0.97	3

This table is based on a full nonlinear regression approach (Supplementary Equation 14) for different taxonomic groups and number of published data on their extinction debt. For estimates of *α* and *k*, we also give the s.d.'s for jackknife replicates (based on source removal). The corresponding sensitivity analyses are given in Supplementary Note 6.
