# Supplementary material for: Dynamics of extinction debt across five taxonomic groups
Source: Nat Commun. 2016 Jul 25;7:12283. doi: 10.1038/ncomms12283 (PMC4962471; doi:10.1038/ncomms12283)
Supplement: Supplementary Information — Supplementary Figures 1-3, Supplementary Table 1, Supplementary Notes 1-6 and Supplementary References [file ncomms12283-s1.pdf]

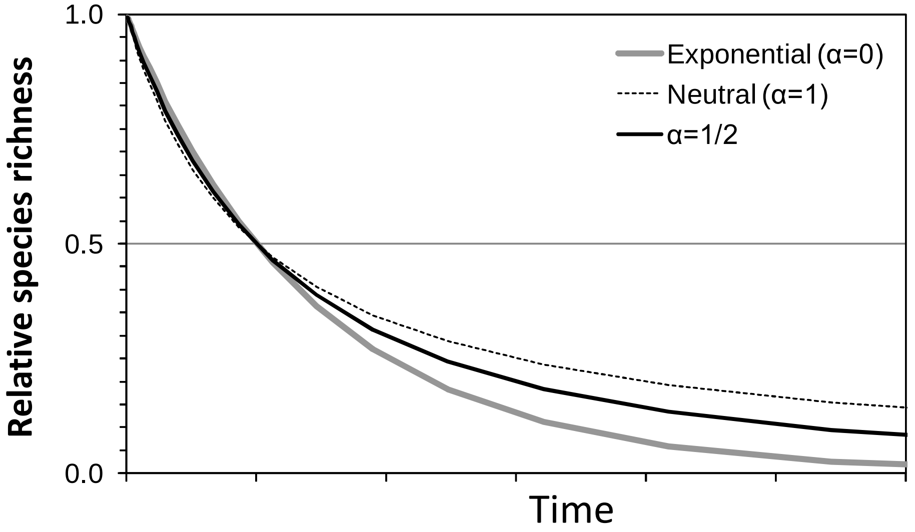

Supplementary Figure 1: Relaxation of relative species richness (*S/S*$_0$) as a function of time. This is given for three different values of parameter α. All curves have the same relaxation time (see Eq. S6).

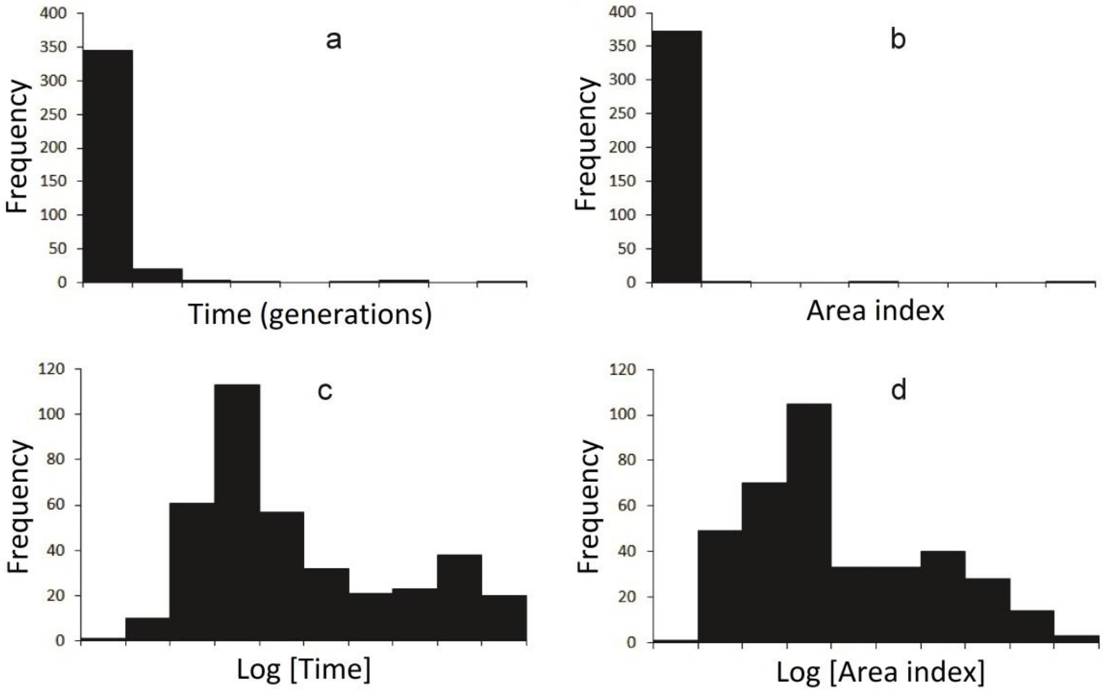

Supplementary Figure 2: Distribution of observed data for time-constant and area index. Histograms showing (**a**) the distribution of observations of T_{50} (estimated half-life) and (**b**) area index, both on linear scales (we note that we define area index as the average number of individuals per species prior to habitat loss). The lower two histograms (**c**) and (**d**) show the corresponding distributions on logarithmic scales. For each histogram, 10 classes were used, equally spaced between the minimum and maximum observations. This illustrates the utility of using logarithmic axes.

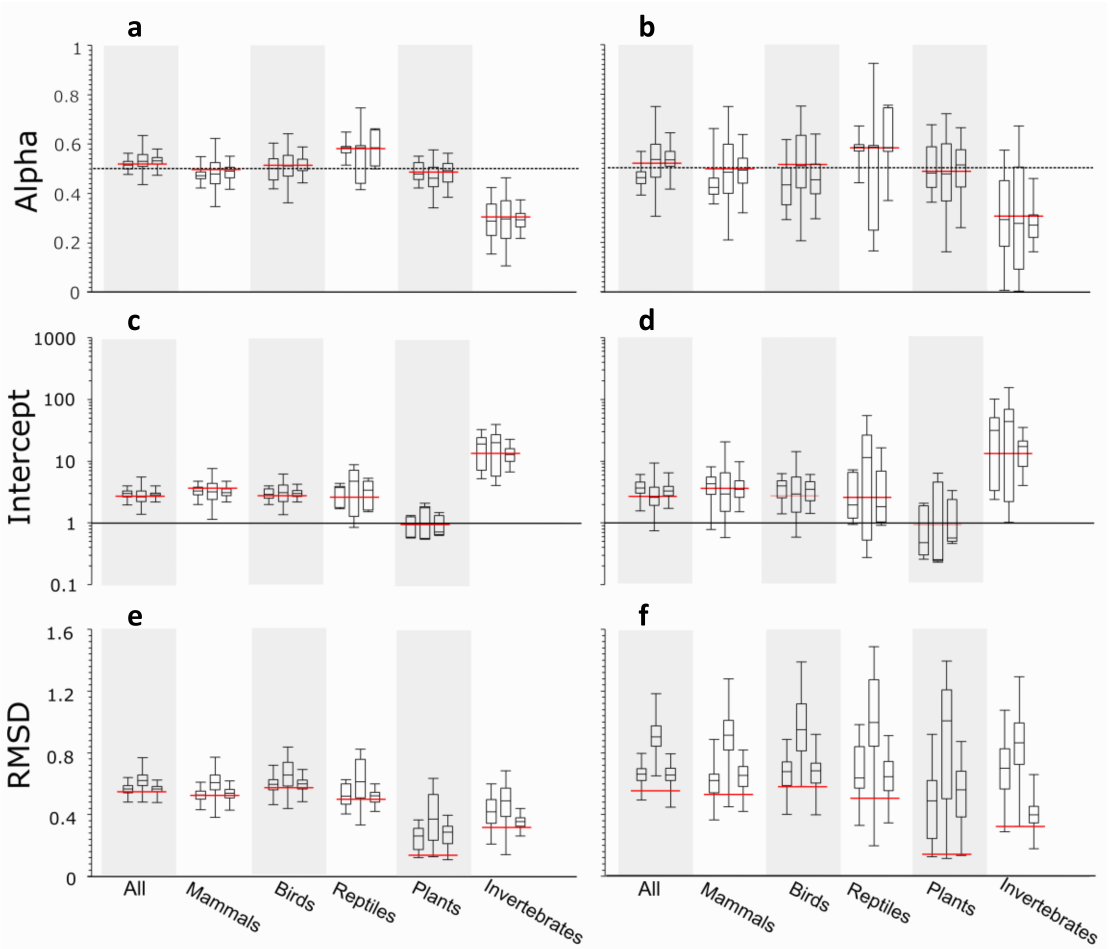

Supplementary Figure 3: Sensitivity analysis. Each box plot describes the range of 200 values for one of the response variables, α, intercept or RMSD, for hypothetical errors in a model parameter. The boxes are arranged in triplets describing the responses to errors, only in parameters τ (left), ρ (middle) and S_0 (right). All left panels (a, c, e) correspond to situations where one of the input parameters is either half as large or twice as large as originally measured or estimated. The panels on the right (b, d, f) are the corresponding responses for even larger errors, where a parameter is either 5 times as large or 1/5 the size. The red lines indicate the levels of the variables originally estimated; in the top panels the level of α=0.5 is marked by a dotted line. For each boxplot, whiskers denote extreme values, central line is the median, and top and bottom of box are the upper and lower 25% levels. The banding is included to improve visibility of graphs.

Supplementary Table 1: List of sources with extinction data. We have arranged sources in an alphabetical order (by first author) but separated those that we have used from those that we have not. Under 'Comments', we describe each source, explain why we have not used it or describe what we did in order to make it usable. For the sources used, if not mentioned in the table, the key parameters, S_0, S_2, Δt or A, are provided. Under 'Taxonomic group' a number in parenthesis indicates that we used this number of sites in our analysis. References within the table are indexed according to the first column, namely ID-1, ID-2, etc.

Source				Taxonomic groups (Number of sites used)	Comments
ID	Ref of Source	Authors	Year		
Publications used					
1	50	D. T. Bolger, A. C. Alberts, R. M. Sauvajot, P. Potenza, C. McCalvin *et al.*	1997	Mammals (25)	Rodents on scrub fragments, in coastal southern California. On the basis of data available[51], we assume that total initial rodent species number, prior to habitat loss, is 21. All estimations of S_2 were by sampling. Of 25 surveyed, 13 fragments had S_2=0.
2	52	J. S. Brashares, P. Arcese, M. K. Sam	2001	Mammals (6)	Large mammals in nature reserves in West Africa. For each reserve, authors provide S_0 based on government publications and S_2 by sampling. Δt represents the time elapsed since the previous study of the area.
3	2	T. M. Brooks, S. L. Pimm, J. O. Oyugi	1999	Birds (5)	Birds in tropical forest fragments, in Kenya. To compute S_0 of each fragment, the authors apply the SAR using the current regional species richness (with z=0.15). Though the habitat was not lost instantaneously, the duration of the loss was short relative to Δt. The authors express confidence that their values for S_2 are proper census, not samples.

4	8	B. W. Brook, N. S. Sodhi, P. K. L. Ng	2003	Birds (1), Mammals (1), Reptiles (1), Invertebrates (1), Amphibians, Fish, Plants	Data for nine taxonomic groups (birds, mammals, amphibians, reptiles, freshwater fish, decapod crustaceans, phasmids, butterflies and vascular plants) on the 2,770 ha of remaining forest in Singapore. We plot data only for those groups whose community size is expected to be proportional to forest area, i.e., birds, mammals, reptiles and butterflies. Decapods are aquatic organisms and it is not so clear what is the loss of wetland area associated with forest loss in Singapore. Same is the problem with phasmids: there is much greater densities of phasmids to be found in the forest boundaries than the forests themselves[53]. Hence losing forest area isn't a clear predictor. S_0 and S_2 for all taxonomic groups that we used were given by the authors based on surveys. For vascular plants, we use the data in ID-38[54].
5	26	J. H. Brown	1971	Mammals (17)	Small boreal mammals on isolated mountaintops in the Great Basin, USA. Author shows that these animals are not distributed as predicted by the equilibrium theory of island biogeography. Time since isolation is end-Pleistocene. S_0 is estimated on the basis of a continental SAR, whereas S_2 on existing surveys and the author's observations over three years.
6	55	M. Castelletta, N. Sodhi, R. Subaraj	2000	Birds (1)	Rainforest birds in Singapore. The country has lost 95% of its native lowland rainforest, mostly in the late-nineteenth century, with only 2,770 ha of primary forest remaining. Both S_0 and S_2 are based on checklists.
7	56	A. G. Chiarello	1999	Mammals (6)	Mammal species of the Atlantic forest, south-eastern Brazil. The authors provide current species numbers on the basis of surveys.
8	57	M. B. Christiansen, E. Pitter	1997	Birds (5)	Birds in forest fragments of south-eastern Brazil. S_0 and S_2 are based on surveys.
9	1	J. M. Diamond	1972	Birds (11)	Pleistocene natural experiment for birds on 16 islands off New Guinea. Diamond assumes 10,000 BP for the time of isolation. For land-bridge islands, he assumes S_0 is the entire mainland fauna. Diamond's estimates of S_2 are proper surveys. We do not analyze 5 small islands, where relaxation seems to be complete.
10	58	J. M. Diamond, K. D. Bishop, S. Van Balen	1987	Birds (1)	Birds in Bogor Botanic Garden, an 86 ha woodland in west Java. Though habitat-loss process was not instantaneous, the duration was short relative to Δt. We know S_0 and S_2 by surveys.
11	39	R. K. Didham, P. M. Hammond, J. H. Lawton, P. Eggleton, N. E. Stork	1998	Invertebrates (3)	Tropical forest beetles, central Amazonia, Brazil. Samples taken in an experimentally fragmented forest landscape. Large dataset collected 10 years after isolation. For extinction, the 29 most common species, found in both fragments and mainland, are considered. S_0 and S_2 are estimated by sampling.

12	59	Z. Ding, K. J. Feeley, Y. Wang, R. J. Pakeman, P. Ding	2013	Birds (41)	Birds on 41 recently-isolated small land-bridge islands in the Thousand Island Lake, China. For S_0, we used the current total species richness of the ensemble. Measurements of S_2 based on samples.
13	48	B. Drayton, R. D. Primack	1996	Plants (1)	Plant species in Middlesex Fells, a 400-ha woodland park in Metropolitan Boston, USA. Authors study changes in species composition between two censuses of the western half, in 1894 and 1993. The Fells are 5 km from nearest protected area, though intervening matrix may provide both refuge and mobility. We do not know the exact time of isolation but assume it is the same as the first time of census.
14	60	K. B. H. Er, J. L. Innes, K. Martin, B. Klinkenberg	2005	Birds (1)	Birds lost from Vancouver, Canada, since spate of urbanization between 1910 and 1930. Original species lists were used in this paper. Authors use the SAR to derive S_0. They don't say much about dispersal and isolation from the surrounding regions. We assume no connection to forest outside the city and all the fragments in city to be connected.
15	61	K. Feeley	2003	Birds (25)	Avian communities on 26 small islands in artificial Lake Guri, Venezuela. Species count on the largest island used as S_0 to generate continental (z=0.15) SAR. Author used sampling, but on the basis of species accumulation curves expresses confidence in the high reliability of the S_2 estimates.
16	3	G. Ferraz, G. J. Russell, P. C. Stouffer, R. O. Bierregaard Jr, S. L. Pimm	2003	Birds (10)	Understory birds in experimentally isolated plots (Biological Dynamics of Forest Fragment Project), central Amazon, Brazil. All species richness values were estimated by sampling. Authors give several estimations of t_{50}. Following Halley and Iwasa[12], we chose the one based on a uniform *a priori* distribution of annual extinction probability.
17	16	L. Gibson, A. J. Lynam, C. J. A. Bradshaw, F. He, D. P. Bickford *et al*	2013	Mammals (26)	Loss of small mammals on 16 of the islands in Chiew Larn artificial reservoir, Thailand. S_2 was estimated by sampling twice, 5-7 and 25-26 years after the creation of the reservoir. We consider S_0 equal to 12, which is the number of species occurring in the area.
18	62	A. Gonzalez	2000	Invertebrates (2)	Micro-arthropod communities on bryophyte-based micro-landscapes, in Derbyshire Peak District, UK. Author presents a time-series of species richness in experimentally created small and large fragments. For each size, S_0 and S_2 are each the average of eight replicate fragments. Species richness is estimated by counting all micro-arthropods after Tullgren-funnel extraction using the 133 identified microarthropod morphotypes as a proxy for S.
19	42	A. Gonzalez, E. J. Chaneton	2002	Invertebrates (2)	Micro-arthropod communities on bryophyte-based micro-landscapes, in Derbyshire Peak District, UK. Data from the same experiment as in ID-18[61].

20	63	T. B. Larsen, K. Aduse-Poku, S. Sáfián	2009	Invertebrates (1)	Butterflies in Boabeng-Fiema Monkey Sanctuary (est. 1984), west Africa. It is uncertain when isolation occurred or how gradual it was. We assume isolation happened midway between 1959, when local villagers realized the problem of deforestation, and 1984, when the area became protected. Authors estimate S_0 equal to 500 butterflies. They found S_2 by a combination of sampling and surveys.
21	64	A.J. Lynam, I. Billick	1999	Mammals	Small mammals in Chiew Larn artificial reservoir, Thailand. This has been superseded by and subsumed in ID-17[16].
22	45	E. G. Leigh Jr, S. J. Wright, E.A. Herre	1993	Plants (6)	Trees on six islands formed about 1913 by the rising waters of Gatun Lake, Panama. Authors count numbers and species of mature trees (dbh≥20cm) on islands, in 1980 and 1989, and on mainland plots of comparable size, in 1980, from which there is an estimate of S_0. The generation time and the number of individuals are also given. For S_2 we use averages between the 1980 and 1989 censuses.
23	65	J. Machunter, W. Wright, R. Loyn, P. Rayment	2006	Birds (4)	Birds in forest remnants, south-eastern Australia. All measurements of species richness were by sampling. Due to the large stochasticity in data, we considered the average species richness for four different classes of area size.
24	66	F. Michalski, C. A. Peres	2007	Mammals (19)	Mammals in Amazonian forest fragments, Brazil. Of the fragments studied by the authors, we used 19 that are almost or entirely isolated from continuous forest. For S_0, we used the total species number of these 19 fragments. S_2 is based on sampling.
25	67	W. D. Newmark	1991	Birds (5)	Understory birds in the Eastern Usambara Mountains, Tanzania. Deforestation between 1885 and the early 1930s led to isolated fragments. Though 10 patches were examined, 6 were in a compact cluster, so we merged them into one. We assume that for all patches S_0=31. S_2 is based on sampling, but it expresses the actual species richness.
26	11	W. D. Newmark	1995	Mammals (10)	Mammals at national parks in western N. America. The pattern of extinction in these parks is consistent with that for land-bridge islands. For S_0 and S_2, park records were used for three separate taxonomic groups: carnivores, artiodactyls, and lagomorphs.
27	68	W. D. Newmark	1996	Mammals (6)	Mammals in national parks of Tanzania. S_0 is known from historical records and S_2 from literature and interviews with resident scientists. Isolation time is assumed to coincide with the time of protection.

28	69	W. D. Newmark, W. T. Stanley, S. M. Goodman	2014	Mammals (7)	Small forest mammals in mountainous forest fragments in the East and West Usambara, Tanzania. For S_0, it was assumed that the control sites reflect the pre-fragmentation conditions for all fragments, while S_2 is based on sampling. Δt is based on historic accounts and interviews with locals.
29	10	A. D. Richman, T. J. Case, T. D. Schwaner	1988	Reptiles (22)	Pleistocene land-bridge study of lizards in Baja California (data from Wilcox, ID-43) and Australia. In contrast to most, these authors supply specific dates for isolation for the two regions, both with many sites. Authors estimate S_0 based on SAR.
30	70	W. D. Robinson	1999	Birds	Birds on Barro Colorado Island, Panama. We have used two studies: ID-31[71] and ID-40[72]. This is subsumed in those.
31	71	W. D. Robinson	2001	Birds (1)	Birds on Barro Colorado Island, Panama. Both S_0 and S_2 are from surveys. S_0 is reported in ID-42[73], ID-30[70] and refers to surveys conducted between 1923 and 1939[74,75]. For this, we used 1930 as a reference year. S_2 is extracted from Table 1 and refers to a survey completed in 1970s[76]. Both counts refer to island resident species including water birds and excluding immigrant and vagrant species.
32	77	N. S. Sodhi, T. M. Lee, L. P. Koh, R. R. Dunn	2005	Birds (1)	Report on the avifaunal turnover in Singapore Botanic Gardens rainforest fragments between 1898 and 1998. Both S_0 and S_2 based on surveys.
33	78	N. S. Sodhi, T. M. Lee, L. P. Koh, D. Prawiradilaga	2006	Birds (1)	Report on avian turnover in Bogor Botanical Gardens, Java. Both S_0 and S_0 based on surveys.
34	79	N. S. Sodhi, D. S. Wilcove, T. M. Lee, C. H. Sekercioglu, R. Subaraj	2010	Birds (4)	Report on the avifaunal turnover on land-bridge islands in south-eastern Asia (Malaysia, Indonesia) following extensive deforestation. S_0 based on surveys, S_2 on sampling. Based on species accumulation curves and observed species richness, authors are confident that their bird inventories are complete.
35	80	M. Soulé, D. T. Bolger, A. C. Alberts, J. Wright, M. Rorice *et al.*	1988	Birds (36)	Study of birds using the same sites in California as in ID-1[50]. Authors present all data that allow calculation of relaxation times, but not the initial bird species number. We estimated S_0 to be 17, which is very close to the total bird species number of the chaparral forests (19) given by the Californian Chaparral Institute[51].
36	9	J. Terborgh	1974	Birds (5)	Birdlife in the Caribbean and eastern Pacific land-bridge islands. Author uses end-Pleistocene for the time of isolation of the land-bridge islands. He notes that they were all once joined to the mainland, thus being continental segments. Therefore, he uses the continental SAR to arrive at values of S_0 for each one. S_2 is based on current surveys.

37	81	J. Terborgh, L. Lopez, J. S. Tello	1997	Birds	Birds on 12 land-bridge islands in Lago Guri, a hydro-electric reservoir, Venezuela. This study is subsumed in ID-15[61].
38	54	I. M. Turner, H. T. W. Tan, Y. C. Wee, Ali Bin Ibrahim, P. T. Chew *et al*.	1994	Plants (1)	Vascular plants of Singapore. Authors report massive extinctions mainly due to deforestation and disturbance. We use the values for forest plants only. Both S_0 and S_2 based on surveys.
39	36	Y. Wang, J. Zhang, K. J. Feeley, P. Jiang, P. Ding	2009	Reptiles (31)	Lizard species inhabiting recently isolated land-bridge islands in the Thousand Island Lake, China.
40	72	D. M. Watson	2010	Birds (1)	Birds on Barro Colorado Island, Panama. Both S_0 and S_2 are from surveys. They refer to island resident species including water birds and excluding immigrant and vagrant species. S_0 is extracted from ID-31[71] and refers to a survey conducted in 1970[76]. S_2 is inferred from Table 2[72] and refers to a survey conducted in 2006.
41	82	E. O. Willis	1974	Birds (1)	Birds on Barro Colorado Island, Panama. We have used two studies: ID-31[71] and ID-40[72]. This is subsumed in those.
42	73	E. O. Willis, E. Eisenmann	1979	Birds	Birds on Barro Colorado Island, Panama. We have used two studies: ID-31[71] and ID-40[72]. This is subsumed in those.
43	83	B. A. Wilcox	1978	Reptiles (17)	Lizards on 17 post-Pleistocene land-bridge islands, in Baja California. This study of Baja islands was re-analyzed in ID-29[10].
Relevant publications not used in our analysis					
44	84	G. H. Adler, J. O. Seamon	1991	Mammals	*Proechirnys semispinosus* populations on islands in Gatun Lake, Panama. Authors examine distribution and abundance patterns on 50 forested islands of only one species, the spiny rat.
45	85	A. Báldi, J. Vörös	2006	Birds, Amphibians, Reptiles	Birds, reptiles and amphibians in Hungarian reserves. Authors compiled species lists for these groups and used the SAR to the whole area of Hungary to derive an estimate of the historical species richness and thus loss of species over the last two centuries. However, the paper does not specify Δt.
46	86	H. Berglund, B. G. Jonsson	2005	Fungi, Lichens	Epiphytic crustose lichens and wood-inhabiting fungi in old-growth forest remnants in boreal Sweden. Authors examine whether species richness in 32 old-growth forest remnants diverges from that in forest patches that have been naturally isolated for millennia. There is no information on Δt or on species richness loss per forest remnant.

47	87	R. Bomarco, R. Lindborg, L. Marini, E.Öckinger	2014	Invertebrates, Plants	Vascular plants and insect taxa collected in grassland fragments of varying size and degree of connectivity in south-eastern Sweden. Information on S_0 is not provided and values of S_2 and A are not explicitly given at the level of individual patches.
48	88	T. C. Bonebrake, D. S. Cooper	2014	Invertebrates	Butterflies in Griffith Park, LA, USA. Authors study the impacts of urbanization on butterfly diversity. The authors compare historical data with records from recent surveys to detect changes in species richness. Data used do not refer to fragments of the park, but to its whole area.
49	89	T. Brooks, A. Balmford	1996	Birds	Birds in Atlantic forest, South America. Authors use the SAR to estimate the endemic bird species that will go extinct due to deforestation. There is no information on S_0 and A loss.
50	90	T. Brooks, J. Tobias, A. Balmford	1999	Birds	Endemic birds of the Atlantic forests, south America. Authors use the SAR to predict future loss of endemic bird species due to deforestation. They compile a dataset of 124 forest-dependent species but they do not provide species richness per patch.
51	91	C. Bulafu, D. Baranga, P. Mucunguzi, R. J. Telford, V.Vandvik	2013	Plants	Composition of 22 forest fragments near Kampala, Uganda. The study is limited to woody plants higher than 3.7 m. After isolation, there continued to be disturbance of unknown magnitude (human interference) for most of the fragments. Isolation seems not to be complete as species richness has even increased in 4 fragments.
52	92	G. R. Canale, C. A. Peres, C. E. Guidorizzi, C. A. Ferreira Gatto, *et al.*	2012	Mammals	Mammals in the Atlantic Forest of north-eastern Brazil. Authors quantify local extinction rates for 10 terrestrial and 7 arboreal midsized to large-bodied mammal species across a large sample of forest patches. No data are provided regarding Δt.
53	93	M. Cannone, S. Pignatti	2014	Plants	Alpine plant communities in the European Alps. Authors examine effects of climate warming on their composition. They present data on the species composition between 1953 and 2003 but there is no reference to A loss.
54	94	G. Ceballos & P.R. Ehrlich	2002	Mammals	Mammal species from six continents. This is a global study on the declines of mammal populations having lost collectively 50% of their historic range. No data are presented on S_0 and Δt.
55	95	Z. Chocholoušková, P.Pyšek	2003	Plants	Flora at the territory of Plzěn city and its surroundings, the Czech Republic. Authors use floristic lists covering a period of 120 years and provide comparisons over three periods of time. They give species numbers but no detailed information on land-use change/habitat loss or fragmentation.

56	96	R. Cintra W.E. Magnusson, A. Albernaz	2013	Birds	Bird assemblages in an Amazonian savannah landscape, Brazil, with forest fragments. There is no information given on Δt, A loss, S_0 and S_2 at the fragment level.
57	97	S. A. O. Cousins	2006	Plants	Plant species in grassland fragments, in different rural landscapes, south-eastern Sweden. Author analyses historical and contemporary grassland patterns and investigates the role of isolation, habitat area, past and present land use and landscape changes. However changes in species richness and A are missing.
58	98	S. A. O. Cousins, D. Vanhoenacker	2011	Plants	Historical and contemporary patterns of plant species in 33 rural landscapes, in south-eastern Sweden. S_0, S_2, Δt are not presented for the individual study sites.
59	99	G. Cowlishaw	1999	Mammals	African forest primates. The author attempts to predict patterns of their decline using SAR under three scenarios of original forest cover and loss. No information is given about S_0, A loss or Δt for the sites occupied by the studied communities.
60	24	G. C. Daily, G. Ceballos, J. Pacheco, G. Suzán, A. Sánchez-Azofeifa	2003	Birds	Non-flying mammals in five habitats of southern Costa Rica, among which forest remnants. Authors note that 6 out of 60 species went extinct in four decades. However, isolation was gradual and certainly not total. Also, the authors provide little information on the fragmentation of the area.
61	100	S. Dullinger, F. Essl, W. Rabitsch, K,-H. Erb, S. Gingrich *et al.*	2013	Mammals, Reptiles, Invertebrates, Plants	Threatened vascular plants, bryophytes, mammals, reptiles, dragonflies and grasshoppers. Authors examine if threats associated with pressures deriving from economic development could be underestimated because of delayed impacts. They test for delays in population declines by relating numbers of threatened species appearing on national red lists of 22 European countries to historical and contemporary levels of socioeconomic pressures. There is no information on Δt and habitat loss and the study areas are too large for a theory geared towards small fragments.
62	101	R. Durães, L. Carrasco, T. B. Smith, J. Karubian	2013	Birds	Understory birds in patches within contiguous forest and in fragments. To examine the effects of the intensity of habitat disturbance/loss, authors compare bird richness across different habitat and landscape types. Data on individual forest fragments are not presented.
63	102	C. J. Ellis, B. J. Coppins	2007	Lichens	Aspen epiphytic lichens, in different habitat types, in Scotland. Authors investigate the relationship between spatial habitat structure and species richness. Although they compare data collected at different habitat units, measured at two spatial scales and for two time frames, it is difficult to extract information on A loss or Δt, while species richness per unit is also lacking.

64	103	F. Escobar, G. Halffter, Á. Solís, V. Halffter, D. Navarrete	2008	Invertebrates	Dung beetle community at La Selva Biological Station, Costa Rica. This has suffered declines in species richness as a result of habitat loss in the surrounding landscape and increasing isolation of the reserve over the last decades. However, isolation is not complete and was never sudden.
65	104	I. C. Fernandez, J. A. Simonetti	2013	Mammals	Small mammal assemblages in the Chilean Mediterranean zone. To explore the effects of urban development, authors estimate species abundance and richness of these animals in urban and rural forest fragments in and close to Santiago. No information is given on Δt and S_0 of the fragments.
66	105	N. R. Franssen, M.Tobler	2013	Fish	Fish assemblage structure in a reservoir in Oklahoma, USA. Authors compare species richness and diversity pre- and post-impoundment and in contemporary streams, above and below the reservoir. No data on species numbers per sampling location are given.
67	106	B. Gilbert, W. F. Laurance, E. G. Leigh Jr, H. E. M. Nascimento	2006	Plants	Tree communities in Brazil. Long-term changes in their composition and dynamics in the study area of the Biological Dynamics of Forest Fragments Project, in central Amazonia, are assessed. Values of Δt are on the order of a decade and so only a fraction of generation times.
68	107	S. M. Goodman, D. Rakotondravony	2000	Mammals	Small mammals (tenrecs) endemic to Madagascar. A survey of this taxon was conducted in a montane forest reserve in the Central High Plateau. Four small fragments and a control site were surveyed. However Δt cannot be established with any confidence.
69	108	C. E. V. Grelle, M. A. S. Alves, H. G. Bergallo, L. Geise, C. F. D. Rocha *et al.*	2005	Birds, Mammals, Amphibians, Reptiles	Birds, mammals, amphibian and reptiles threatened with extinction, in Rio de Janeiro State, Brazil. Authors test the effect of deforestation on them. Data are not provided by fragment. Getting the original data for S_0, S_2 and A requires original sources in Portuguese, which are not readily accessible.
70	109	W. Gu, R. Heikkilä, I. Hanski	2002	Fungi	Polyporous fungi in boreal forests in eastern Finland. Authors apply a metapopulation model to simulate the occurrence of species in a landscape that has been subjected to habitat loss and fragmentation. They focus their analyses on only four species of threatened polyporous fungi, which does not allow us to extract information on S_0.
71	110	M. Guardiola, J. Pino, F. Rodà	2013	Plants	Mediterranean grasslands in southern Catalonia. Authors investigate the effect of habitat loss on plant species richness. They provide full list of plants in all study sites but not the species number per patch examined.

72	111	I. Hanski, E. Koivulehto, A. Cameron, P. Rahagalala	2007	Invertebrates	Endemic, forest-dwelling Helictopleurini dung beetles in Madagascar. Authors investigate the effect of forest loss on this taxon. There is no information by fragment; all results are grouped together. There is also no indication of fragment *A* or Δt.
73	112	J. S. Harding, E. F. Benfield, P. V. Bolstad, G. S. Helfman, E. B. D. Jones	1998	Fish, Invertebrates	Stream invertebrates and fish in two river basins, in western North Carolina, USA. Authors investigate the influence of past land use on the present day diversity of these taxa and conclude that preservation of habitat fragments may not be sufficient to maintain natural diversity in streams. There is no information on S_0, *A*, or Δt.
74	113	T. Heinken, E. Weber	2013	Plants	Endangered plant species in Germany. This is a meta-analysis, in which authors examine whether species differing in phylogeny, habitat requirements, and biology are likely to respond differently to habitat fragmentation. There is no information on Δt, *A* loss, S_0 or S_0.
75	114	A. Helm, I. Hanski, M. Pärtel	2006	Plants	Vascular plant species in calcareous grasslands of Estonia. Authors compare 35 grassland patches of different size and explore the response of plants to long-term habitat loss and fragmentation. No information is given on Δt and S_0.
76	115	S. Highland, J. A. Jones	2014	Invertebrates, Plants	Plants and nocturnal moths in natural montane meadows in Oregon, USA. Authors explore impacts of habitat loss and fragmentation on these taxa. They reconstruct the actual size of each studied meadow, but they do not provide information on the size of sampling units and on the number of species in them.
77	116	G. Hu, K. J. Feeley, J. Wu, G. Xu, M. Yu	2011	Plants	Vascular plants in the Thousand Island Lake in southeast China. Authors collected data from 154 islands to explore factors that determine plant species richness and patterns of species nestedness. There is no island-specific information on *A*, S_0 or S_2.
78	117	I. Husáková, Z. Münzbergová	2014	Plants	Plants at dry, grassland-like forest openings, in Czech Republic. Authors explore the importance of past and present landscape structures and habitat conditions on species richness. They do not provide data on S_0, S_2 and *A* for the 110 forest openings that they studied.
79	118	V. Johansson, T. Snäll, T. Ranius	2013	Lichens	Oak epiphytic lichens in southeast Sweden. Authors examine whether current or historical landscape structure can explain present-day occurrence patterns. No data on *A*, S_0 or S_2 per sampling unit (tree) are presented.

80	119	M. Kopecký, R. Hédl, P. Szabó	2013	Plants	Forest plant community at Děvín, the Czech Republic. Authors analyse temporal changes after the mid-twentieth-century abandonment of coppicing in a typical Central European forest. The paper is structured towards detecting changes in plant composition due to changes of the management scheme rather than due to isolation. So, A loss and Δt are not given.
81	120	G. Kattan, H. Alvarez-López, M. Giraldo	1994	Birds	Forest bird species in a fragmented cloud-forest site in the western Andes, Colombia. Authors compare occurrence data collected in 1911 and 1959 with data collected from a 1989-1990 survey. No information on historic or current A of the studied fragments is provided.
82	121	M. Laaksonen, E. Peuhu, G. Várkonyi, J. Siitonen	2008	Invertebrates, Fungi	Wood-decomposing fungi and saproxylic beetles inhabiting patches of spruce-swamp forests of Finland and Russia. The authors investigate the effect of habitat quality, loss and isolation on richness patterns. Patch-level information is not given.
83	122	T.B. Larsen	2008	Invertebrates	Forest butterflies in Africa, west of the Dahomey Gap. Authors report that 97% of all species recorded from the area are still present despite the fact that during the past 150 or so years the forests shrunk to 13% or less of their original extent. There is little information on Δt and remaining areas are too large (100,000 km^2 in total) for a theory geared towards small fragments.
84	123	H. Läattman, K.-O. Bergman, M. Rapp, M.Täalle, L. Westerberg *et al.*	2014	Lichens	Oak epiphytic lichens in urban and rural areas, southeast Sweden. Authors examine how urbanization affects abundance and richness. There is no information given on species richness per sampling unit and on Δt.
85	124	W. F. Laurance	1991	Mammals	Non-flying mammals in tropical Queensland, Australia. To explore the vulnerability of tropical forest biota to habitat fragmentation, the author tests the efficacy of ecological traits to predict responses of these animals. The paper does not provide clear information on A, Δt or S_2 on a per site basis.
86	125	A. C. Lees, C. A. Peres	2006	Birds	Tropical forest avifauna, southern Amazonia, Brazil. Authors sampled disturbed and undisturbed primary forest patches of various sizes to study the effects of fragmentation upon avian community structure. A and S_2 for each fragment are not provided.
87	126	R. Lindborg	2007	Plants	Plants in semi-natural grasslands, in southeast Sweden. Author examines the effects of land-use change on plant species composition over time. There is a list of all plants recorded within the 25 grassland sites investigated, but there is no information on S_0 and on the current and historical A of these sites.

88	127	R. Lindborg, O. Eriksson	2004	Plants	Plants in semi-natural grasslands in southeast Sweden. Authors investigate the temporal scale of species response to landscape change. Remnants of traditionally managed semi-natural grasslands were sampled and their species diversity was related to current and past (50-100 yrs) landscape connectivity properties. As above, there is no information given on S_0 and A of each sampling unit.
89	128	J. L. McCune, M.Vellend	2013	Plants	Plant communities on southern Vancouver island, Canada. Authors explore how disturbance affects plant communities in human-dominated landscapes. Surveying plots repeatedly, they examine changes in species diversity over time. There is no information on species richness per plot. In addition, despite the severe disturbance due to urbanization over the past 40 years, there are striking increases in species richness indicating high levels of colonization.
90	129	P. I. Olivier, R. van Aarde, A. T. Lombard	2013	Birds	Birds in KwaZulu-Natal province, South Africa. Authors examine if habitat suitability models can be used in conjunction with SAR to estimate extinction debt implied by the conservation status of extant species. The model was parameterised by using information on historic distributions of coastal forests that have suffered extensive loss. There is no information on Δt, S_0, S_2 or actual A loss per fragment.
91	130	M. Őster, S. A. O. Cousins, O. Eriksson	2007	Plants	Grassland plants in southern Sweden. To examine if plant diversity in fragmented semi-natural grasslands is related to present and historical landscape context, measured as connectivity, authors surveyed 30 sites of different size. No information on S_0, A loss or Δt is provided.
92	131	H. Paltto, B. Nordén, F. Götmark, N. Franc	2006	Plants, Lichens, Fungi	Red Data Book and Indicator Species of plants, lichens and wood-inhabiting fungi in old temperate broadleaved forests in southern Sweden. Authors test the relative effect of different spatial and temporal scales for occurrence and persistence of species of conservation concern. There is no clear information on temporal scales, fragmentation or isolation history, as well as on species richness and A per sampling unit.
93	132	M. Pärtel, R. Mändla, M. Zobel	1999	Plants	Plants of the largest calcareous semi-natural grassland in Estonia. To investigate the importance of landscape history in determining vegetation patterns, authors carried out a vegetation survey. They distinguish and compare ancient grasslands, grasslands on former arable fields and forest clear-cut areas in the study site. However, there is no information given on A loss, S_0 or S_2 at the fragment level.

94	133	M. Pärtel, A. Helm, T. Reitalu, J. Liira, M. Zobel	2007	Plants	Vascular plants in calcareous grasslands in Estonia. Authors explore the relationship between plant diversity and both current and past human population density, during the last iron age. No information is provided on S_0, S_2, Δt or A loss.
95	134	Penttilä, M.Lindgren, O. Miettinen, H. Rita, I. Hanski	2006	Fungi	Polypores and other wood-decomposing fungi in forest fragments, in Finland. To explore the species sensitivity to habitat loss and fragmentation, authors compare data from two regions with contrasting histories of forestry and marked differences in the amount and spatial configuration of old-growth forests. There is no information on S_0.
96	135	K. Piessens, M. Hermy	2006	Plants	Heathland and forest plant species in north-western Belgium. Using historical plant distribution data, authors examine how the loss of heathland area has affected heathland and forest plant communities, and if heathland flora shows an extinction debt. There is no information given on Δt or reduction of A at the fragment level.
97	136	H. Piha, M. Luoto, J. Merila	2007	Amphibians	Amphibians on the island of Gotland, south-eastern Sweden. To examine the importance of current and historic land-use patterns in determining wetland species distributions, authors analyzed data on the presence of amphibian species at sites in Gotland, which has lost more than 40% of its wetlands since the 18th century. There is no information given on species richness or Δt at the fragment level.
98	137	S. L. Pimm, R. A. Askins	1995	Birds	Birds of eastern North America. Authors analyse the distribution of bird species and the timing and extent of forest loss. The paper applies to continental size areas and, therefore, it is not suitable for extracting data for this study.
99	138	E. T. Ranius, P. Eliasson, P. Johansson	2008	Lichens, Fungi	Red-listed lichen and fungal species on old oaks, in southeast Sweden. Current occurrence patterns of these taxa were selected to investigate any association with the current and historical density of big oaks and connectivity properties of the landscape. There is no information given on S_0 or A loss.
100	139	L. M. Renjifo	1999	Birds	Avifauna in sub-Andean Colombia. Author assesses the effects of long-term fragmentation by comparing the occurrence of bird species in forest fragments isolated over 50-90 years with the original avifauna. There is no information on S_2 and A per fragment.

101	140	R. Ribon, J. E. Simon, G. T. de Mattos	2003	Birds	Atlantic forest birds in south-eastern Brazil. Authors study their conservation status in forest fragments of different size so as to assess the effects of forest fragmentation and destruction on them. There is no information given on *A*, S_0 and S_2 at the fragment level.
102	141	V. A. O. Selonen, J. S. Kotiaho	2013	Plants	Vascular plants and mosses at the riparian habitat of small boreal streams, in Finland. Authors compare community composition in sites at mature spruce-dominated forests having a distance from the stream from 0 to 50 m. The temporal component associated with differences in participating species is the time since the adjacent forest was harvested, which varied from 1 to 50 years. However, this cannot be considered as equivalent to Δt. Also, species richness per site examined is not provided.
103	142	K. E. Sieving	1992	Birds	Birds on Barro Colorado Island (BCI), Panama. Author examines five insectivorous species testing the hypothesis that interspecific variation in nest design and placement underlies differential avian extinction from BCI. We have used two studies for birds from BCI: ID-31[71] and ID-40[72].
104	143	B. J. Sigel, T. W. Sherry, B. E. Young	2006	Birds	Birds at La Selva Biological Station, Costa Rica. Given the gradual conversion of most of the tropical forest surrounding the station to agricultural land since the 1960s, authors mainly focus on changes in the bird-community composition via changes in abundance. There is no specific information given on the number of species lost or Δt.
105	144	M. Soga, S. Koike	2012	Invertebrates	Butterflies in Tokyo, Japan. Authors examine the relative importance of habitat quantity, quality and isolation on butterfly assemblages in urban forest fragments. It is not possible to estimate S_0 nor Δt.
106	145	M. Soga, S. Koike	2013	Invertebrates	Butterflies in Tokyo, Japan. Here, authors compare the effects of current and past landscape parameters on current species richness and explore the relationship between life-history traits and the extent of extinction debts in a modern city. As for ID-105[144], it is not possible to estimate S_0 nor Δt for each fragment.
107	25	P. C. Stouffer, C. Strong, L. N. Naka	2009	Birds	Understorey birds in the Amazonian rainforest, Brazil. Authors studied the same system as Ferraz et *al.*[3], but later. In this phase, the process of extinction has slowed and is matched by recolonization, possibly due to the re-growth of forests in-between fragments. For our model, in which recolonization is assumed to be zero, these data are not appropriate.

108	146	K. W. Thijs, R. Aerts, W. Musila, M. Siljander, E. Matthysen *et al.*	2014	Plants	Tree species in 12 remaining Afromontane cloud forest relics in Taita Hills, Kenya. Authors introduce a species accounting equation, which includes various ecological processes and allows evaluation of the current forest composition and assessment of potential future dynamics. To solve it, they use data derived from a single inventory. There is no information given on S_0 and Δt.
109	147	I. M. Turner, K. S. Chua, J. S. Y. Ong, B. C. Soong, H. T. W. Tan	1996	Plants	Vascular plants in Singapore Botanic Gardens. Authors compare species composition with the historic record of the flora of the Gardens' jungle obtained from the extensive collection of herbarium specimens dating back to the 1890s. Although 228 historically recorded native species are not present any more, there are 94 new native species in the contemporary flora indicating strong immigration effects.
110	148	D. Vallan	2000	Amphibians	Amphibian diversity in a nature reserve, highland Madagascar. S_0 is from census while all S_2 are samples. Although there is evidence that fragments may be old, most of the deforestation has been recent. Δt is not given and A is confounded by the fact that some fragments contain more water than others.
111	149	S. Van der Veken, K. Verheyhen, M. Hermy	2004	Plants	Plant species in Turnhout, Belgium. Authors study species loss in this urban area comparing records from 1880s to 1990s. They report that the most important factors involved in changes in plant diversity and vegetation composition are habitat loss due to urbanization and habitat deterioration, mainly due to agricultural intensification. No details are given for the heathland fragments while analysis is done on the scale of 1 km^2 cells that may or may not be isolated.
112	150	M. Vellend, K. Verheyen, H. Jacquemyn, A. Kolb, H. Van Calster *et al.*	2006	Plants	Woodland vascular plants in Lincolnshire, UK and Vlaams-Brabant, Belgium. Authors select 36 species common to both sites to compare observed and predicted occupancy patterns of forest patches on the basis of species history traits. There is no information on Δt, A loss and species richness at the patch level.
113	151	K. D. Wagner, J. Krauss, I. Steffan-Dewenter	2013	Invertebrates	Butterflies along an altitudinal gradient in a low mountain region, in Bavaria, Germany. Authors study the effects of altitude and historical land cover change on butterfly diversity. Authors provide detailed lists of historical and current records, but the sites studied are considered as parts of a continuous mosaic rather than isolated patches.

114	152	M. Yu, G. Hu, K. J. Feeley, J. Wu, P. Ding	2012	Birds, Plants	Plants and birds on 41 islands in Thousand Island Lake, China. Authors explore whether the effects of habitat fragmentation on species richness are taxon and/or scale dependent. There is no indication of S_0, whereas S_2 are merged for all islands.
115	153	Y. Basset, H. Barrios, S. Segar, R. B. Srygley, A. Aiello *et al*.	2015	Invertebrates	The butterflies of Barro Colorado Island, Panama: local extinction since the 1930s. Authors use published lists and recent monitoring programs to evaluate changes in butterfly composition on BCI between old (1923 – 1943) and recent (1993 –2013) periods. They present data in the form of nine categories (including new and cryptic species and species of unclear status), which was difficult to express in terms of our framework.

Supplementary Notes

Supplementary Note 1: Population-based model of species loss from a fragment

The theoretical model for the loss of biodiversity in a community describes the process, where an area of size A_0 suddenly contracts to size A, and species richness, in response, declines from S_0 to a new equilibrium value S (Fig. 1). Just prior to habitat loss, the system is assumed to be in equilibrium. Habitat loss is assumed to be sudden, complete and permanent, which means that there is no life supported in the matrix between islands[24], no re-growth of forest[25] and no restoration of habitat. It is also assumed that the area of the habitat remnant is much smaller than the initial area and that subsequent changes in its size or isolation are negligible. We ignore explicit spatial structure of the habitat or in the pattern of extinctions. We assume species to have the same population density, that immigration and speciation do not play an important role in this process, that the community is well-mixed and that species are not clustered. Time is measured in generations. Each time the model is applied, the species considered are assumed to have the same generation time.

A widely used equation for species loss can be put in a biogeographical framework as follows:

$$\frac{dS}{dt} = I - eS \tag{S1}$$

The first term on the right-hand side of the equation, I, is the rate of colonization (Immigration) by new species per unit time. The second term is the corresponding rate of extinction. We assume that the habitat remnant contains a number of individuals, J, proportional to the area: $J=\rho A$ (A is the area of the habitat remnant, and ρ is the density of individuals per unit area) over all S species (initially S_0). We also assume that the probability of extinction of each species decreases with increasing population size, n, specifically that the probability of extinction per generation, e, is inversely proportional to the number of individuals per species raised to some power $\alpha \geq 0$:

$$e = k\left(\frac{1}{n}\right)^{\alpha} = k\left(\frac{J}{S}\right)^{-\alpha} \tag{S2}$$

We ignore the spectrum of population sizes of the different species. The form above is quite general. The constant α determines whether decreases in population increase extinction probability strongly (large α) or weakly (small α). Eq. S1 thus becomes:

$$\frac{dS}{dt} = I - k\left(\frac{S}{\rho A}\right)^{\alpha} S, \quad S(0)=S_0 \tag{S3}$$

The first term on the right-hand side in the above equation is associated with speciation and colonization. This means that Eq. S3 includes natural turnover (extinction and recolonization or speciation) as well as extinctions due to interventions. The form for the solution of S3 for general values of α can be found by integrating directly, but the closed-form solution is not simple. However, in cases

where immigration or speciation can be considered relatively small, we can assume I=0. We have then Eq. 1 (main text), for which the solution is quite simple:

$$S(t) = \frac{S_0}{\sqrt[\alpha]{1 + k\alpha \left(\frac{S_0}{\rho A} \right)^{\alpha} t}} = \frac{S_0}{\sqrt[\alpha]{1 + \frac{k\alpha t}{n_0^{\alpha}}}} \quad , \quad \alpha > 0 \qquad \text{(S4)}$$

Here, we have used Eq. 1, $n_0 = \rho A / S_0$. This extinction curve is strongly non-exponential. For most values of α, the form is closer to hyperbolic. Its non-exponential character is more pronounced for large α. The solution above does, however, converge to exponential when $\alpha \to 0$ (extinction-probability independent of population size), for which the solution is $S = S_0 \text{Exp}(-kt)$.

An important special case is α=1/2, which is associated with a decay of the form:

$$S(t) = \frac{S_0}{\left[1 + \frac{kt}{2\sqrt{n_0}} \right]^2} \quad \sim \quad \frac{2S_0}{k^2} t^{-2} \quad (for \quad t \to \infty) \qquad \text{(S5)}$$

Thus, for large values of time, the decay in species richness takes the form of a power law, which for this special case is proportional to $1/t^2$.

This model assumes that all species are equal. Some (neutral) variability could be added[12], which might offer some extra insights into the range of variability of relaxation time, but this would require a greater level of sophistication to solve the stochastic differential equation. As was found in earlier work[12], the locus of the average is more or less the same. On the other hand, systematic trait-based variability between species is still a topic of ongoing research and there is not yet a consensus on how this kind of variability will affect these models.

Supplementary Note 2. Time constants

The first time-constant of relaxation is the expected time for half the extinction debt to be paid off. In this, as in earlier studies[12], we assume an absence of speciation and colonization (I=0). Thus, for the model we adopt, the half-life of extinction debt is equal to the time for species richness to fall to half its original value. This is calculated by letting $S = S_0/2$ in Eq. S4 above, from which we get Eq. 2 in the text. In terms of n_0, this is Eq. 6 in text:

$$t_{50} \quad = \quad \frac{2^{\alpha} - 1}{\alpha k} \cdot n_0^{\alpha} \qquad \text{(S6)}$$

From Eq. S4 we can also estimate the expected time for the extinction of the first species ($S_0(t_F) = S_0 - 1$):

$$t_F = \frac{t_{50}}{(2^{\alpha} - 1) S_0} = \frac{n_0^{\alpha}}{\alpha k S_0} \qquad \text{(S7)}$$

Similarly, for the extinction of all but the last species ($S(t_L)=1$), the time is given by:

$$t_L = \frac{S_0^\alpha - 1}{2^\alpha - 1} t_{50} \tag{S8}$$

Supplementary Note 3. Connection with the continental species-area relationship

The diversity capacity of larger areas is greater, a fact which is reflected in the species-area relationship (SAR), which often takes the Arrhenius form $S = cA^z$. Suppose we do not know the initial number of species, S_0, but would like an *a priori* forecast of the three time constants in terms of the true area (as opposed to area index). Then, our only option is to use the SAR. If the SAR for the system is $S_0 = cA^z$, we can write t_{50} using (S6) as:

$$t_{50} = \frac{2^\alpha - 1}{k\alpha}\left(\frac{\rho A}{cA^z}\right)^\alpha = \frac{2^\alpha - 1}{(c/\rho)^\alpha k\alpha} A^{\alpha - z\alpha} \tag{S9}$$

and the time until first determined extinction as:

$$t_F = \frac{\rho^\alpha A^\alpha}{k\left(cA^z\right)^{\alpha+1}} = \frac{(\rho/c)^\alpha}{ck} A^{\alpha - z\alpha - z} \tag{S10}$$

Similarly, the time for extinction of all but the last species is:

$$t_L = (S_0^\alpha - 1)(n_0^\alpha / k) = \frac{S_0^\alpha - 1}{2^\alpha - 1} t_{50} \approx S_0^\alpha n_0^\alpha / k \approx \frac{\rho^\alpha}{k\alpha} A^\alpha \tag{S11}$$

The exponent z is usually small for continental SARs. For example, Brown[26] derives the formula for small mammals: $S = 5.68A^{0.121}$ (with area in hectares). We can derive a continental SAR for birds in tropical forests from the species richness of New Guinea[1], S_0=518 with A=7.86×10^7 ha, and then use the exponent z=0.15 to give the equation $S = 33.6A^{0.15}$. These parameter values are used in Fig. 3 in the main text.

Supplementary Note 4. Finding parameters α and k from data

For each habitat remnant, along with location and taxonomic information for the species involved, we have a set of values for the following variables:

$$\left\{S_0, S_2, \Delta t, A, \rho, \tau\right\}_j$$

Given this set of records, our goal is to find the constants α and k from them. This is done through Eq. S4 following the approach used in Halley & Iwasa[12]. The first step is to express Eq. S4 in terms of the half-life, t_{50}, using Eq. S6 to get:

$$S(t) = \frac{S_0}{\sqrt[\alpha]{1 + \left(2^\alpha - 1\right)\frac{t}{t_{50}}}} \tag{S12}$$

We can rearrange this equation as follows:

$$t_{50}(\alpha) = \frac{2^{\alpha} - 1}{(S_0 / S(t))^{\alpha} - 1}\left(\frac{t}{\tau}\right) \tag{S13}$$

The species richness $S(t)$ in our model is only an expected value, whereas a real observation $S_2(t)$ may be affected by environmental variability and measurement error: $S_2(t) = S(t) + \varepsilon(t)$. Thus, when we substitute our observed species number $S_2(t)$ into Eq. S13, we have the *estimated half-life*, T_{50}, which is Eq. 7 in text:

$$T_{50}(\alpha) = \frac{2^{\alpha} - 1}{(S_0 / S_2)^{\alpha} - 1}\left(\frac{\Delta t}{\tau}\right)$$

Our approach here is based on that of Halley & Iwasa[12], in which T_{50} was compared with t_{50} so as to show that $T_{50} \approx t_{50}$ and hence to argue that the neutral model could be a reasonable approximation of reality. In this case, however, our aim is to estimate the best values of the model parameters α and k, by minimizing the difference between t_{50} and T_{50}. Thus, we minimize the mean-square difference between $\ln t_{50}$ and $\ln T_{50}$, namely we minimize the quantity $\sum \ln(T_{50} / t_{50})^2$ over the possible range of α and k:

$$\min\left\{\sum_j \left[\ln\left[\frac{\alpha k(\Delta t_j / \tau_j)}{(S_{0j} / S_{2j})^{\alpha} - 1} \Big/ \left(\frac{\rho_j A_j}{S_{0j}}\right)^{\alpha}\right]\right]^2\right\}_{\alpha,k} \tag{S14}$$

To carry out the calculations for this nonlinear regression, we employed Excel macros using the Solver tool to obtain minimum values. The fitting is done in the logarithmic domain because there is a large range of scales involved. For example, area ranges from less than one hectare to hundreds of square kilometers, whereas time, Δt, ranges from a few years to millennia. This can be seen in the histograms of n_0 and T_{50} on linear and logarithmic scales (Fig. S2). It is clear that for log scales the data are distributed relatively evenly along the axis for both parameters, whereas on linear scales, they are concentrated towards the origin. It is clear that for representation and for regression the log scale will be more meaningful.

The analogy with linear regression is also clear when we apply a log transformation (and let $b = \ln(\alpha k)$):

$$\sum_j \left[\ln\left[\frac{\Delta t_j / \tau_j}{(S_{0j} / S_{2j})^{\alpha} - 1}\right] - \ln\left[\left(\frac{\rho_j A_j}{S_{0j}}\right)^{\alpha} \cdot (\alpha k)\right]\right]^2$$
$$= \sum_j \left[\ln T_{50j}(\alpha) - \alpha \ln n_{0j} - b(\alpha, k)\right]^2 \qquad \ldots\text{(S15)}$$

However, unlike in linear regression, here we don’t have a simple graphical interpretation because the constant b of the regression and T_{50} are themselves functions of α and k.

For the neutral case (α=1), this estimated relaxation time is Eq. 9 in text:

$$T_{50}(1) = \frac{\Delta t / \tau}{S_0 / S_2 - 1}$$

Although we do not assume a neutral model, this $T_{50}(1)$ is the half-life of the equivalent neutral community. If we approximate the time T_{50j} by the neutral half-life[12] $T_{50j}(1)$, then we have the following minimization problem:

$$\min\left\{ \sum_j \left[\ln T_{50\,j}(1) - \alpha \ln n_{0\,j} - b(\alpha,k) \right]^2 \right\}_{\alpha,k} \qquad \text{(S16)}$$

This can be solved by simple linear regression. Thus, if $T_{50j}(\alpha) \approx T_{50j}(1)$, then (S16) gives us values of k and α, not too different from the values that minimize (S14). Even if $T_{50j}(\alpha) \neq T_{50j}(1)$, in most cases, the values of k and α will not change very much. In a best-fit regression, the points $T_{50j}(\alpha)$ will lie above and below the regression line. The effect of decreasing α is to decrease the variability around this value but there is no dependence on n_{0j}. Thus, while changing α will affect the positions of individual data points relative to the average, it will have little effect on the average itself. Therefore, the area dependence in Eq. S15 tends to be dominated by the middle term $\alpha \ln(n_{0j})$, which is the same in both S14 and S16.

Supplementary Note 5. Sources of data

In order to assemble observations of real extinction events, we carried out an extensive search of the existing literature. It is difficult to locate all sources of information on the subject since many relevant papers have been published over the years without keywords connecting them to extinction debt or relaxation time. To find sources of data, we performed standard internet-based searches, made use of existing large compilations[4,6,12] and drew on our own archives. A surprising number of sources could only be located after checking references of individual papers.

Of the papers that we examined, we include in Supplementary Table 1 papers providing data relevant to the loss of biodiversity that follows habitat loss. We separated sources that we have used form those that we have not. For a literature source to be used in our analysis, it should provide or allow calculation of the following four parameters: (i) initial species richness (S_0), (ii) final species richness (S_2), (iii) remaining final area (A), and (iv) time-span between the act of isolation and the time when the species present were determined (Δt). It should also satisfy the following three requirements: (a) the act of isolation it refers to was reasonably sudden, (b) there is complete isolation of the remaining fragments from the main area, and (c) there is no regeneration. Violation of these assumptions is the usual reason why literature sources are excluded from our analysis. For each of the sources included in Supplementary Table 1, we explain what we did in order to make it usable (if applicable) or why we have not used it.

Supplementary Note 6. Analysis of sensitivity

On the way to the main result, Eq. 4 in the text, all of the measured or inferred parameters, S_0, S_2, ρ, A, Δt and τ, are subject to uncertainty. In addition to actual measurement inaccuracy, errors are likely to arise also through two major assumptions in our model. First is the assumption of abrupt losses of area, when it might have happened in stages. The second is that we are modelling an ensemble of different species with a single value of ρ or τ. Could the main results actually be dependent on an erroneous value? If so, our result would be overturned once we had better measurements. To answer this question, we carry out the following sensitivity analysis.

(i) Parameters ρ and A. From Eq. S13, it is clear that if either ρ or A is Q times smaller, the error undergoes an addition of $\alpha \ln Q$. Thus, ρ or A have the same effect. The area A is reasonably straightforward to measure. Errors arise mainly through ρ, which is often poorly known or poorly defined. Such is the case with butterflies, but it is also problematic for herbaceous plants in a mixed environment. As most studies are carried out for a single taxonomic group, investigations of errors in ρ should also be at the level of the study.

(ii) Parameters τ and Δt. If Δt is Q times larger or smaller, then the error term has an addition of $\ln Q$. Thus, the error is the same for Δt and τ differing only in sign. The time elapsed (Δt) is usually relatively well known. For invertebrates, generation times τ are frequently unknown. For larger species, the problem is more likely to lie with the fact that we are modelling an ensemble of species with a single generation time, something that is not fully remedied by altering an average value. As most studies are carried out for a single taxonomic group, investigations of errors in τ, as for ρ, should be at the level of the study.

(iii) Parameters S_0 and S_2. The main problem here is that when $S_0 \approx S_2$, attempting to describe errors by multiplying by Q or $1/Q$ could cause Eq. S13 to yield a senseless answer. However, we know that $S_0 \geq S_2$ in all cases. Thus, it makes more sense to consider ΔS times Q or $1/Q$ in each case. In general, the effect of errors in S_2 is not exactly the same as for S_0. However, because usually the errors in S_0 are much greater than those in S_2, since S_2 is measured directly but S_0 must be inferred or guessed, we will only consider errors in S_0. Thus, for ΔS to change by a factor Q, we require S_0 to change by a factor of R_0, which can be calculated as follows:

$$Q\Delta S = R_0 S_0 - S_2 \Rightarrow R_0 = Q + (1-Q) S_2 / S_0 \tag{S17}$$

Thus, our task can be reduced to the analysis of three parameters instead of six: ρ, τ and S_0. For each taxonomic group (and the ensemble), we tested the influence of two quite large levels of error (Q=2 and Q=5) in each of the three chosen parameters. For all observations in each study in our dataset, we changed the value of the parameter in question at random to either Q or $1/Q$ of its original value. We then generated 200 simulations per examined combination (parameter, error magnitude, taxonomic group) finding the median, hinges and extremes for α, intercept and the RMSD (from F. S14) each time.

As expected, well-represented groups (mammals and birds) have narrower intervals. Groups with few studies (reptiles) or few points (invertebrates) have wider intervals. We see more non-uniform behaviour in groups like reptiles and plants that feature large numbers of points from a small number of studies. The exponent α remains fairly robust, even in the face of very large errors, especially for mammals and birds. The outlier status of the invertebrates remains even with large errors assumed. For the intercept, invertebrates and plants are outliers and remain so. Groups with few studies (reptiles and plants) or few points (invertebrates) have much wider intervals. Note that RMSD values are lower for plants and invertebrates but this is probably fortuitous. This difference disappears with larger error levels. For the RMSD, errors in ρ have a greater effect on the responses than errors in τ or S_0.

Supplementary References

1 Diamond, J. M. Biogeographic kinetics: estimation of relaxation times for avifaunas of southwest Pacific islands. *Proc. Natl. Acad .Sci .USA* **69**, 3199-3203 (1972).

2 Brooks, T. M., Pimm, S. L. & Oyugi, J. O. Time lag between deforestation and bird extinction in tropical forest fragments. *Conserv. Biol.* **13**, 1140-1150 (1999).

3 Ferraz, G. *et al.* Rates of species loss from Amazonian forest fragments. *Proc. Natl. Acad .Sci .USA* **100**, 14069-14073 (2003).

4 Kuussaari, M. *et al.* Extinction debt: a challenge for biodiversity conservation. *Trends Ecol. Evol* **24**, 564-571 (2009).

5 Tilman, D., May, R. M., Lehman, C. L. & Nowak, M. A. Habitat destruction and the extinction debt. *Nature* **371**, 65 - 66 (1994).

6 Wearn, O. R., Reuman, D. C. & Ewers, R. M. Extinction debt and windows of conservation opportunity in the Brazilian Amazon. *Science* **337**, 228-232 (2012).

7 Pimm, S. L. & Raven, P. Biodiversity: extinction by numbers. *Nature* **403**, 843-845 (2000).

8 Brook, B. W., Sodhi, N. S. & Ng, P. K. Catastrophic extinctions follow deforestation in Singapore. *Nature* **424**, 420-426 (2003).

9 Terborgh, J. Preservation of natural diversity: the problem of extinction prone species. *Bioscience* **24**, 715-722 (1974).

10 Richman, A. D., Case, T. J. & Schwaner, T. D. Natural and unnatural extinction rates of reptiles on islands. *Am. Nat.* **131**, 611-630 (1988).

11 Newmark, W. D. Extinction of mammal populations in western North American national parks. *Conserv. Biol.* **9**, 512-526 (1995).

12 Halley, J. M. & Iwasa, Y. Neutral theory as a predictor of avifaunal extinctions after habitat loss. *Proc. Natl. Acad .Sci .USA* **108**, 2316-2321 (2011).

13 Hubbell, S. P. *The unified neutral theory of biodiversity and biogeography (MPB-32).* Vol. 32 (Princeton University Press, 2001).

14 Ricklefs, R. E. The unified neutral theory of biodiversity: do the numbers add up? *Ecology* **87**, 1424-1431 (2006).

15 Clark, J. S. The coherence problem with the Unified Neutral Theory of Biodiversity. *Trends Ecol. Evol* **27**, 198-202 (2012).

16 Gibson, L. *et al.* Near-complete extinction of native small mammal fauna 25 years after forest fragmentation. *Science* **341**, 1508-1510 (2013).

17 Hylander, K., Ehrlén, J. The mechanisms causing extinction debts. *Trends Ecol. Evol.* **28**, 341-346 (2013).

18 Halley, J. M., Iwasa, Y. & Vokou, D. Comment on" Extinction debt and windows of conservation opportunity in the Brazilian Amazon". *Science* **339**, 271-271 (2013).

19 Wearn, O. R., Reuman, D. C. & Ewers, R. M. Response to Comment on" Extinction debt and windows of conservation opportunity in the Brazilian Amazon". *Science* **339**, 271-271 (2013).

20 Kitzes, J. & Harte, J. Predicting extinction debt from community patterns. *Ecology* **96**, 2127–2136 (2015).

21 Hanski, I. Extinction debt and species credit in boreal forests: modelling the consequences of different approaches to biodiversity conservation. *Ann. Zool. Fenn.* **37**, 271-280 (2000).

22 Dirzo, R. *et al.* Defaunation in the Anthropocene. *Science* **345**, 401-406 (2014).
23 Pimm, S. L. *et al.* The biodiversity of species and their rates of extinction, distribution, and protection. *Science* **344**, 1246752 (2014).
24 Daily, G. C., Ceballos, G., Pacheco, J., Suzán, G. & Sánchez, A., Arturo. Countryside biogeography of neotropical mammals: conservation opportunities in agricultural landscapes of Costa Rica. *Conserv. Biol.* **17**, 1814-1826 (2003).
25 Stouffer, P. C., Strong, C. & Naka, L. N. Twenty years of understorey bird extinctions from Amazonian rain forest fragments: consistent trends and landscape-mediated dynamics. *Divers. Distrib.* **15**, 88-97 (2009).
26 Brown, J. H. Mammals on mountaintops: nonequilibrium insular biogeography. *Am. Nat.* **105**, 467-478 (1971).
27 Myers, P., R. Espinosa, C. S. Parr, T. Jones, G. S. Hammond, T. A. Dewey. The Animal Diversity Web (http://animaldiversity.org) (2015).
28 Millar, J. S. & Zammuto, R. M. Life histories of mammals: an analysis of life tables. *Ecology* **64**, 631-635 (1983).
29 Eisenberg, J. F. in *Conservation Biology: An Evolutinary-Ecological Perspective* (ed B.S. Wilcox M.E. Soulé) 35-55 (Sinauer Press, Sunderland, Massachuesetts, 1980).
30 Caro, T. Densities of mammals in partially protected areas: the Katavi ecosystem of western Tanzania. *J. Appl. Ecol.* **36**, 205-217 (1999).
31 Chiarello, A. G. Density and population size of mammals in remnants of Brazilian Atlantic forest. *Conserv. Biol.* **14**, 1649-1657 (2000).
32 Mares, M. A. & Ernest, K. A. Population and community ecology of small mammals in a gallery forest of central Brazil. *J. Mammal.* **76**, 750-768 (1995).
33 Parmenter, R. R. *et al.* Small-mammal density estimation: a field comparison of grid-based vs. web-based density estimators. *Ecol. Monogr.* **73**, 1-26 (2003).
34 Hairston, N. G. *Vertebrate zoology: an experimental field approach*. (CUP Archive, 1994).
35 Buckley, L. B. & Jetz, W. Insularity and the determinants of lizard population density. *Ecol. Lett.* **10**, 481-489 (2007).
36 Wang, Y., Zhang, J., Feeley, K., Jiang, P. & Ding, P. Life-history traits associated with fragmentation vulnerability of lizards in the Thousand Island Lake, China. *Anim. Conserv.* **12**, 329-337 (2009).
37 Grøtan, V., Lande, R., Engen, S., Sæther, B. E. & DeVries, P. J. Seasonal cycles of species diversity and similarity in a tropical butterfly community. *J. Anim. Ecol.* **81**, 714-723 (2012).
38 Weimers, M. *personal communication* (2015).
39 Didham, R. K., Hammond, P. M., Lawton, J. H., Eggleton, P. & Stork, N. E. Beetle species responses to tropical forest fragmentation. *Ecol. Monogr.* **68**, 295-323 (1998).
40 Nakamura, K., Hasan, N., Abbas, I., Godfray, H. C. J. & Bonsall, M. B. Generation cycles in Indonesian lady beetle populations may occur as a result of cannibalism. *Proc.R. Soc. Biol. Sci. Ser. B* **271**, S501-S504 (2004).
41 Novotny, V. M. *personal communication* (2015).
42 Gonzalez, A. & Chaneton, E. J. Heterotroph species extinction, abundance and biomass dynamics in an experimentally fragmented microecosystem. *J. Anim. Ecol.* **71**, 594-602 (2002).

43 Carrillo, D., Peña, J. E., Hoy, M. A. & Frank, J. H. Development and reproduction of Amblyseius largoensis (Acari: Phytoseiidae) feeding on pollen, Raoiella indica (Acari: Tenuipalpidae), and other microarthropods inhabiting coconuts in Florida, USA. *Exp. Appl. Acarol.* **52**, 119-129 (2010).
44 Chen, Y. Microarthropod diversity and distribution in Southwestern Canada. (2013). MSc thesis, Univ. British Columbia (2013)
45 Leigh Jr, E. G., Wright, S. J., Herre, E. A. & Putz, F. E. The decline of tree diversity on newly isolated tropical islands: a test of a null hypothesis and some implications. *Evol. Ecol.* **7**, 76-102 (1993).
46 Poulsen, A. D. & Balslev, H. Abundance and cover of ground herbs in an Amazonian rain forest. *J. Veg. Science* **2**, 315-322 (1991).
47 Poulsen, A. D. *The herbaceous ground flora of the Batu Apoi forest Reserve, Brunei Darussalam* (Springer, 1996).
48 Drayton, B. & Primack, R. B. Plant species lost in an isolated conservation area in metropolitan Boston from 1894 to 1993. *Conserv. Biol.* **10**, 30-39 (1996).
49 Pearson, S. M., Smith, A. B. & Turner, M. G. Forest patch size, land use, and mesic forest herbs in the French Broad River Basin, North Carolina. *Castanea* **63**, 382-395 (1998).
50 Bolger, D. T. *et al.* Response of rodents to habitat fragmentation in coastal southern California. *Ecol. Appl.* **7**, 552-563 (1997).
51 Quinn, R. D. & Keeley, S. C. *Introduction to California chaparral*. Vol. 90 (Univ of California Press, 2006).
52 Brashares, J. S., Arcese, P. & Sam, M. K. Human demography and reserve size predict wildlife extinction in West Africa. *ProC.R. Soc. Biol. Sci. Ser. B* **268**, 2473-2478 (2001).
53 Berger, J. *Ecology of phasmids (Phasmatodea) in a moist neotropical forest: a study on life history, host range and bottom up versus top down regulation* Dissertation zur Erlangung des Naturwissenschafttlichen Doktorgrades der Technischen Universität Kaiserslautern thesis, Kaiserslautern (2004).
54 Turner, I. *et al.* A study of plant species extinction in Singapore: lessons for the conservation of tropical biodiversity. *Conserv. Biol.* **8**, 705-712 (1994).
55 Castelletta, M., Sodhi, N. S. & Subaraj, R. Heavy extinctions of forest avifauna in Singapore: lessons for biodiversity conservation in Southeast Asia. *Conserv. Biol.* **14**, 1870-1880 (2000).
56 Chiarello, A. G. Effects of fragmentation of the Atlantic forest on mammal communities in south-eastern Brazil. *Biol. Conserv.* **89**, 71-82 (1999).
57 Christiansen, M. B. & Pitter, E. Species loss in a forest bird community near Lagoa Santa in southeastern Brazil. *Biol. Conserv.* **80**, 23-32 (1997).
58 Diamond, J. M., Bishop, K. D. & Balen, S. v. Bird survival in an isolated Javan woodland: island or mirror? *Conserv. Biol.* **1**, 132-142 (1987).
59 Ding, Z., Feeley, K. J., Wang, Y., Pakeman, R. J. & Ding, P. Patterns of bird functional diversity on land-bridge island fragments. *J. Anim. Ecol.* **82**, 781-790 (2013).
60 Er, K. B. H., Innes, J. L., Martin, K. & Klinkenberg, B. Forest loss with urbanization predicts bird extirpations in Vancouver. *Biol. Conserv.* **126**, 410-419 (2005).
61 Feeley, K. Analysis of avian communities in Lake Guri, Venezuela, using multiple assembly rule models. *Oecologia* **137**, 104-113 (2003).

62 Gonzalez, A. Community relaxation in fragmented landscapes: the relation between species richness, area and age. *Ecol. Lett.* **3**, 441-448 (2000).
63 Larsen, T., Aduse-Poku, K. & Sáfián, S. The butterflies of Boabeng-Fiema Monkey Sanctuary-biodiversity and extinction in a forest fragment in Ghana. *Afr. Entomol.* **16**, 131-146 (2009).
64 Lynam, A. & Billick, I. Differential responses of small mammals to fragmentation in a Thailand tropical forest. *Biol. Conserv.* **91**, 191-200 (1999).
65 MacHunter, J., Wright, W., Loyn, R. & Rayment, P. Bird declines over 22 years in forest remnants in southeastern Australia: Evidence of faunal relaxation? *Can. J. For. Res.* **36**, 2756-2768 (2006).
66 Michalski, F. & Peres, C. A. Disturbance-mediated mammal persistence and abundance-area relationships in Amazonian forest fragments. *Conserv. Biol.* **21**, 1626-1640 (2007).
67 Newmark, W. D. Tropical forest fragmentation and the local extinction of understory birds in the Eastern Usambara Mountains, Tanzania. *Conserv. Biol.* **5**, 67-78 (1991).
68 Newmark, W. D. Insularization of Tanzanian parks and the local extinction of large mammals. *Conserv. Biol.* **10**, 1549-1556 (1996).
69 Newmark, W. D., Stanley, W. T. & Goodman, S. M. Ecological correlates of vulnerability to fragmentation among Afrotropical terrestrial small mammals in northeast Tanzania. *J. Mammal.* **95**, 269-275 (2014).
70 Robinson, W. D. Long-term changes in the avifauna of Barro Colorado Island, Panama, a tropical forest isolate. *Conserv. Biol.* **13**, 85-97 (1999).
71 Robinson, W. D. Changes in abundance of birds in a Neotropical forest fragment over 25 years: a review. *Anim. Biodivers. Conserv.* **24**, 51-65 (2001).
72 Watson, D. M. Optimizing inventories of diverse sites: insights from Barro Colorado Island birds. *Methods Ecol. Evol.* **1**, 280-291 (2010).
73 Willis, E. O., E. Eisenmann A revised list of the birds of Barro Colorado Island. Panama. *Smithson Contrib Zool* **291**, 1-31 (1979).
74 Chapman, F. M. *My tropical air castle*. (Appleton-Century, New York 1929).
75 Chapman, F. M. *Life in an air castle*. (Appleton-Century, New York 1938).
76 Willis, E. Ecological roles of migratory and resident birds on Barro Colorado Island, Panama. *Migrant birds in the Neotropics: ecology, behavior, distribution and conservation*, 205-225 (1980).
77 Sodhi, N. S., Lee, T. M., Koh, L. P. & Dunn, R. R. A century of avifaunal turnover in a small tropical rainforest fragment. *Anim. Conserv.* **8**, 217-222 (2005).
78 Sodhi, N. S., Lee, T. M., Koh, L. P. & Prawiradilaga, D. M. Long-term avifaunal impoverishment in an isolated tropical woodlot. *Conserv. Biol.* **20**, 772-779 (2006).
79 Sodhi, N. S. et al. Deforestation and avian extinction on tropical landbridge islands. *Conserv. Biol.* **24**, 1290-1298 (2010).
80 Soulé, M. E. et al. Reconstructed dynamics of rapid extinctions of chaparral-requiring birds in urban habitat islands. *Conserv. Biol.* **2**, 75-92 (1988).
81 Terborgh, J., Lopez, L. & Tello S, J. Bird communities in transition: the Lago Guri islands. *Ecology* **78**, 1494-1501 (1997).
82 Willis, E. O. Populations and local extinctions of birds on Barro Colorado Island, Panama. *Ecol. Monogr.* **44**, 153-169 (1974).
83 Wilcox, B. A. Supersaturated island faunas: a species-age relationship for lizards on post-Pleistocene land-bridge islands. *Science* **199**, 996-998 (1978).

84 Adler, G. H. & Seamon, J. O. Distribution and abundance of a tropical rodent, the spiny rat, on islands in Panama. *J. Trop. Ecol.* **7**, 349-360 (1991).

85 Báldi, A. & Vörös, J. Extinction debt of Hungarian reserves: a historical perspective. *Basic Appl. Ecol.* **7**, 289-295 (2006).

86 Berglund, H. & Jonsson, B. G. Verifying an extinction debt among lichens and fungi in northern Swedish boreal forests. *Conserv. Biol.* **19**, 338-348 (2005).

87 Bommarco, R., Lindborg, R., Marini, L. & Öckinger, E. Extinction debt for plants and flower-visiting insects in landscapes with contrasting land use history. *Divers. Distrib.* **20**, 591-599 (2014).

88 Bonebrake, T. C. & Cooper, D. S. A Hollywood drama of butterfly extirpation and persistence over a century of urbanization. *J. Insect Conserv.* **18**, 683-692 (2014).

89 Brooks, T. & Balmford A. Atlantic forest extinctions. *Nature* **380**, 115 (1996).

90 Brooks, T., Tobias, J. & Balmford, A. Deforestation and bird extinctions in the Atlantic forest. *Anim. Conserv.* **2**, 211-222 (1999).

91 Bulafu, C., Baranga, D., Mucunguzi, P., Telford, R. & Vandvik, V. Massive structural and compositional changes over two decades in forest fragments near Kampala, Uganda. *Ecol. Evol.* **3**, 3804-3823 (2013).

92 Canale, G. R., Peres, C. A., Guidorizzi, C. E., Gatto, C. A. F. & Kierulff, M. C. M. Pervasive defaunation of forest remnants in a tropical biodiversity hotspot. *PLoS ONE* **7***:* e41671 (2012).

93 Cannone, N. & Pignatti, S. Ecological responses of plant species and communities to climate warming: upward shift or range filling processes? *Clim. Change* **123**, 201-214 (2014).

94 Ceballos, G. & Ehrlich, P. R. Mammal population losses and the extinction crisis. *Science* **296**, 904-907 (2002).

95 Chocholoušková, Z. & Pyšek, P. Changes in composition and structure of urban flora over 120 years: a case study of the city of Plzeň. *Flora* **198**, 366-376 (2003).

96 Cintra, R., Magnusson, W. E. & Albernaz, A. Spatial and temporal changes in bird assemblages in forest fragments in an eastern Amazonian savannah. *Ecol. Evol.* **3**, 3249-3262 (2013).

97 Cousins, S. A. Plant species richness in midfield islets and road verges–the effect of landscape fragmentation. *Biol. Conserv.* **127**, 500-509 (2006).

98 Cousins, S. A. & Vanhoenacker, D. Detection of extinction debt depends on scale and specialisation. *Biol. Conserv.* **144**, 782-787 (2011).

99 Cowlishaw, G. Predicting the pattern of decline of African primate diversity: an extinction debt from historical deforestation. *Conserv. Biol.* **13**, 1183-1193 (1999).

100 Dullinger, S. *et al.* Europe's other debt crisis caused by the long legacy of future extinctions. *Proc. Natl. Acad .Sci .USA* **110**, 7342-7347 (2013).

101 Durães, R., Carrasco, L., Smith, T. B. & Karubian, J. Effects of forest disturbance and habitat loss on avian communities in a Neotropical biodiversity hotspot. *Biol. Conserv.* **166**, 203-211 (2013).

102 Ellis, C. J. & Coppins, B. J. 19th century woodland structure controls stand-scale epiphyte diversity in present-day Scotland. *Divers. Distrib.* **13**, 84-91 (2007).

103 Escobar, F., Halffter, G., Solís, Á., Halffter, V. & Navarrete, D. Temporal shifts in dung beetle community structure within a protected area of tropical

wet forest: a 35-year study and its implications for long-term conservation. *J. Appl. Ecol.* **45**, 1584-1592 (2008).
104 Fernández, I. C. & Simonetti, J. A. Small mammal assemblages in fragmented shrublands of urban areas of Central Chile. *Urban Ecosyst.* **16**, 377-387 (2013).
105 Franssen, N. & Tobler, M. Upstream effects of a reservoir on fish assemblages 45 years following impoundment. *J. Fish Biol.* **82**, 1659-1670 (2013).
106 Gilbert, B., Laurance, W. F., Leigh Jr, E. G. & Nascimento, H. E. Can Neutral Theory predict the responses of Amazonian tree communities to forest fragmentation?. *Am. Nat.* **168**, 304-317 (2006).
107 Goodman, S. M. & Rakotondravony, D. The effects of forest fragmentation and isolation on insectivorous small mammals (Lipotyphla) on the Central High Plateau of Madagascar. *J. Zool.* **250**, 193-200 (2000).
108 Grelle, C. *et al.* Prediction of threatened tetrapods based on the species–area relationship in Atlantic Forest, Brazil. *J. Zool.* **265**, 359-364 (2005).
109 Gu, W., Heikkilä, R. & Hanski, I. Estimating the consequences of habitat fragmentation on extinction risk in dynamic landscapes. *Landsc. Ecol.* **17**, 699-710 (2002).
110 Guardiola, M., Pino, J. & Rodà, F. Patch history and spatial scale modulate local plant extinction and extinction debt in habitat patches. *Divers. Distrib.* **19**, 825-833 (2013).
111 Hanski, I., Koivulehto, H., Cameron, A. & Rahagalala, P. Deforestation and apparent extinctions of endemic forest beetles in Madagascar. *Biol. Lett.* **3**, 344-347 (2007).
112 Harding, J., Benfield, E., Bolstad, P., Helfman, G. & Jones, E. Stream biodiversity: the ghost of land use past. *Proc. Natl. Acad .Sci .USA* **95**, 14843-14847 (1998).
113 Heinken, T. & Weber, E. Consequences of habitat fragmentation for plant species: Do we know enough? *Perspect. Plant. Ecol. Evol. Syst.* **15**, 205-216 (2013).
114 Helm, A., Hanski, I. & Pärtel, M. Slow response of plant species richness to habitat loss and fragmentation. *Ecol. Lett.* **9**, 72-77 (2006).
115 Highland, S. A. & Jones, J. A. Extinction debt in naturally contracting mountain meadows in the Pacific Northwest, USA: varying responses of plants and feeding guilds of nocturnal moths. *Biodivers. Conserv.* **23**, 2529-2544 (2014).
116 Hu, G., Feeley, K. J., Wu, J., Xu, G. & Yu, M. Determinants of plant species richness and patterns of nestedness in fragmented landscapes: evidence from land-bridge islands. *Landsc. Ecol.* **26**, 1405-1417 (2011).
117 Husáková, I. & Münzbergová, Z. Relative importance of current and past landscape structure and local habitat conditions for plant species richness in dry grassland-like forest openings. *PLoS ONE* **9***:* e97110 (2014).
118 Johansson, V., Snäll, T. & Ranius, T. Estimates of connectivity reveal non-equilibrium epiphyte occurrence patterns almost 180 years after habitat decline. *Oecologia* **172**, 607-615 (2013).
119 Kopecký, M., Hédl, R. & Szabó, P. Non-random extinctions dominate plant community changes in abandoned coppices. *J. Appl. Ecol.* **50**, 79-87 (2013).
120 Kattan, G. H., Alvarez-López, H. & Giraldo, M. Forest fragmentation and bird extinctions: San Antonio eighty years later. *Conserv. Biol.* **8**, 138-146 (1994).

121 Laaksonen, M., Peuhu, E., Várkonyi, G. & Siitonen, J. Effects of habitat quality and landscape structure on saproxylic species dwelling in boreal spruce-swamp forests. *Oikos* **117**, 1098-1110 (2008).
122 Larsen, T. B. Forest butterflies in West Africa have resisted extinction… so far (Lepidoptera: Papilionoidea and Hesperioidea). *Biodivers. Conserv.* **17**, 2833-2847 (2008).
123 Lättman, H. *et al.* Decline in lichen biodiversity on oak trunks due to urbanization. *Nord. J. Bot.* **32**, 518-528 (2014).
124 Laurance, W. F. Ecological correlates of extinction proneness in Australian tropical rain forest mammals. *Conserv. Biol.* **5**, 79-89 (1991).
125 Lees, A. C. & Peres, C. A. Rapid avifaunal collapse along the Amazonian deforestation frontier. *Biol. Conserv.* **133**, 198-211 (2006).
126 Lindborg, R. Evaluating the distribution of plant life-history traits in relation to current and historical landscape configurations. *J. Ecol.* **95**, 555-564 (2007).
127 Lindborg, R. & Eriksson, O. Historical landscape connectivity affects present plant species diversity. *Ecology* **85**, 1840-1845 (2004).
128 McCune, J. L. & Vellend, M. Gains in native species promote biotic homogenization over four decades in a human-dominated landscape. *J. Ecol.* **101**, 1542-1551 (2013).
129 Olivier, P. I., Aarde, R. J. & Lombard, A. T. The use of habitat suitability models and species–area relationships to predict extinction debts in coastal forests, South Africa. *Divers. Distrib.* **19**, 1353-1365 (2013).
130 Öster, M., Cousins, S. A. & Eriksson, O. Size and heterogeneity rather than landscape context determine plant species richness in semi-natural grasslands. *J.Veg. Science* **18**, 859-868 (2007).
131 Paltto, H., Nordén, B., Götmark, F. & Franc, N. At which spatial and temporal scales does landscape context affect local density of Red Data Book and Indicator species? *Biol. Conserv.* **133**, 442-454 (2006).
132 Pärtel, M., Mändla, R. & Zobel, M. Landscape history of a calcareous (alvar) grassland in Hanila, western Estonia, during the last three hundred years. *Landsc. Ecol.* **14**, 187-196 (1999).
133 Pärtel, M., Helm, A., Reitalu, T., Liira, J. & Zobel, M. Grassland diversity related to the Late Iron Age human population density. *J. Ecol.* **95**, 574-582 (2007).
134 Penttilä, R., Lindgren, M., Miettinen, O., Rita, H. & Hanski, I. Consequences of forest fragmentation for polyporous fungi at two spatial scales. *Oikos* **114**, 225-240 (2006).
135 Piessens, K. & Hermy, M. Does the heathland flora in north-western Belgium show an extinction debt? *Biol. Conserv.* **132**, 382-394 (2006).
136 Piha, H., Luoto, M. & Merilä, J. Amphibian occurrence is influenced by current and historic landscape characteristics. *Ecol. Appl.* **17**, 2298-2309 (2007).
137 Pimm, S. L. & Askins, R. A. Forest losses predict bird extinctions in eastern North America. *Proc. Natl. Acad .Sci .USA* **92**, 9343-9347 (1995).
138 Ranius, T., Eliasson, P. & Johansson, P. Large-scale occurrence patterns of red-listed lichens and fungi on old oaks are influenced both by current and historical habitat density. *Biodivers. Conserv.* **17**, 2371-2381 (2008).
139 Renjifo, L. M. Composition Changes in a Subandean Avifauna after long-term forest fragmentation. *Conserv. Biol.* **13**, 1124-1139 (1999).

140 Ribon, R., Simon, J. E. & Theodoro De Mattos, G. Bird extinctions in Atlantic forest fragments of the Viçosa region, southeastern Brazil. *Conserv. Biol.* **17**, 1827-1839 (2003).
141 Selonen, V. A. & Kotiaho, J. S. Buffer strips can pre-empt extinction debt in boreal streamside habitats. *BMC Ecol.* **13**, 24 (2013).
142 Sieving, K. E. Nest predation and differential insular extinction among selected forest birds of central Panama. *Ecology* **73**, 2310-2328 (1992).
143 Sigel, B. J., Sherry, T. W. & Young, B. E. Avian community response to lowland tropical rainforest isolation: 40 years of change at La Selva Biological Station, Costa Rica. *Conserv. Biol.* **20**, 111-121 (2006).
144 Soga, M. & Koike, S. Relative importance of quantity, quality and isolation of patches for butterfly diversity in fragmented urban forests. *Ecol. Res.* **27**, 265-271 (2012).
145 Soga, M. & Koike, S. Mapping the potential extinction debt of butterflies in a modern city: implications for conservation priorities in urban landscapes. *Anim. Conserv.* **16**, 1-11 (2013).
146 Thijs, K. W. *et al.* Potential tree species extinction, colonization and recruitment in Afromontane forest relicts. *Basic Appl. Ecol.* **15**, 288-296 (2014).
147 Turner, I., Chua, K., Ong, J., Soong, B. & Tan, H. A century of plant species loss from an isolated fragment of lowland tropical rain forest. *Conserv. Biol.* **10**, 1229-1244 (1996).
148 Vallan, D. Influence of forest fragmentation on amphibian diversity in the nature reserve of Ambohitantely, highland Madagascar. *Biol. Conserv.* **96**, 31-43 (2000).
149 Van der Veken, S., Verheyen, K. & Hermy, M. Plant species loss in an urban area (Turnhout, Belgium) from 1880 to 1999 and its environmental determinants. *Flora* **199**, 516-523 (2004).
150 Vellend, M. *et al.* Extinction debt of forest plants persists for more than a century following habitat fragmentation. *Ecology* **87**, 542-548 (2006).
151 Wagner, K. D., Krauss, J. & Steffan-Dewenter, I. Butterfly diversity and historical land cover change along an altitudinal gradient. *J. Insect Conserv.* **17**, 1039-1046 (2013).
152 Yu, M., Hu, G., Feeley, K. J., Wu, J. & Ding, P. Richness and composition of plants and birds on land-bridge islands: effects of island attributes and differential responses of species groups. *J. Biogeogr.* **39**, 1124-1133 (2012).
153 Basset, Y., Barrios, H., Segar, S., Srygley, R.B., Aiello, A., Warren, A.D., Delgado, F., Coronado, J., Lezcano, J., Arizala, S. & Rivera, M. 2015. The butterflies of Barro Colorado Island, Panama: local extinction since the 1930s. *PloS ONE,* **10**, e0136623.
